# Spatial Transcriptome and Single Nucleus Transcriptome Sequencing Reveals Tetrahydroxy Stilbene Glucoside Promotes Ovarian Organoids Development Through the Vegfa‐Ephb2 Pair

**DOI:** 10.1002/advs.202410098

**Published:** 2024-12-04

**Authors:** Chunlan Mu, Xiaoyong Li, Jiamei Yang, Geng G. Tian, Hepeng Bai, Wenhui Lin, Linhui Wang, Ji Wu

**Affiliations:** ^1^ School of Basic Medical Sciences Key Laboratory of Fertility Preservation and Maintenance of Ministry of Education Ningxia Medical University Yinchuan 750004 China; ^2^ Key Laboratory for the Genetics of Developmental & Neuropsychiatric Disorders (Ministry of Education) Bio‐X Institutes Shanghai Jiao Tong University Shanghai 200240 China; ^3^ School of Pharmacology Ningxia Medical University Yinchuan 750004 China; ^4^ School of Agriculture and Biology Shanghai Jiao Tong University Shanghai 200240 China; ^5^ School of Life Sciences and Biotechnology Joint International Research Laboratory of Metabolic and Developmental Sciences Shanghai Jiao Tong University Shanghai 200240 China; ^6^ Department of Urology, Changhai Hospital Second Military Medical University (Naval Medical University) Shanghai 200433 China; ^7^ Shanghai Key Laboratory of Cell Engineering Shanghai 200433 China

**Keywords:** ligand‐receptor pairs, ovarian organoids, plant‐derived compounds, single nucleus transcriptome sequencing, spatial transcriptome sequencing

## Abstract

Ovarian dysfunction is a major factor leading to female infertility. Understanding how to improve or reshape ovarian function has become an important entry point for preventing and treating female infertility caused by ovarian dysfunction. Here, plant‐derived compounds are screened for in vitro activity upon ovarian organoids derived from feeder‐free female germline stem cells. Tetrahydroxy stilbene glucoside (TSG) is found to promote the development of ovarian organoids. Single nucleus transcriptome sequencing and spatial transcriptome sequencing are used to establish a comprehensive spatiotemporal map to elucidate the role of TSG in ovarian organoid development, encompassing cell types and subtypes, transcription factors, pseudo‐time sequence, and cell communication dynamics. This analysis indicates that TSG promotes ovarian organoid development through the vascular endothelial growth factor A‐Eph receptor B2 ligand‐receptor pair between granulosa cells and oocytes. This study has enhanced the understanding of the mechanisms of ovarian organoid development, establishes a technical platform for screening compounds for treating infertility and related diseases, and lays a foundation for clinically applying plant‐derived compounds.

## Graphical abstract

The screening of plant‐derived compounds on ovarian organoids derived from feeder‐free female germline stem cells finds that tetrahydroxy stilbene glucoside (TSG) promotes follicle development and steroid hormone synthesis. Mechanistically, ST‐seq and snRNA‐seq reveal that TSG promotes ovarian organoid development through the vascular endothelial growth factor A‐Eph receptor B2 ligand‐receptor pair between granulosa cells and oocytes.

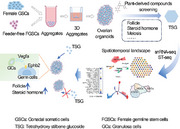

## 1 Introduction

Infertility is a major health issue, with 17% of the global population affected.^[^ ^1^ ^]^ Abnormal biological processes, including organogenesis, meiosis, hormone production and regulation, and mitosis, contribute to the occurrence and high frequency of infertility.^[^ ^2^ ^]^ Among the risk factors of female infertility, ovarian dysfunction is one of the main drivers leading to the failure of oogenesis.^[^ ^2^ ^]^ For example, ≈1% of women under 40 years of age experience primary ovarian insufficiency.^[^ ^3^ ^]^ Despite the high and rising prevalence of ovarian dysfunction, effective treatments for this condition are lacking. Treating ovarian dysfunction will be important for intervention in female infertility.

Organoid technology has generated a powerful model to research ovarian development in vitro. In recent decades, relevant in vitro models of ovarian organoids or reconstituted ovaries have been established using induced pluripotent stem cells, embryonic stem cells, and female germline stem cells (FGSCs).^[^ ^4^, ^5^, ^6^, ^7^, ^8^, ^9^, ^10^ ^]^ Our laboratory has used FGSCs to generate ovarian organoids, which exhibited key features of ovaries in vivo, including oogenesis and steroidogenesis, and ultimately produced oocytes leading to the successful production of offspring.^[^ ^9^ ^]^ Ovarian organoids can also serve as effective experimental models for toxicology analysis, fluorescent semiconducting polymer dot‐based delivery of nucleic acid, and pharmacological screening.^[^ ^9^, ^11^, ^12^ ^]^ Therefore, ovarian organoids may provide a suitable model for studying potential factors for the treatment of ovarian dysfunction.

Recent studies have reported that phytochemicals extracted from plants, such as *C. sativus*, *Angelica sinensis*, *Paeonia lactiflora*, *Pueraria lobata*, *Magnoliae Flos*, and *Chrysin*, promoted ovarian development, and further suggested that plant‐derived compounds may have therapeutic potential in regard to ovarian dysfunction.^[^ ^13^, ^14^, ^15^, ^16^, ^17^, ^18^ ^]^ However, in other research on plant‐derived compounds, therapeutic efficacies were evaluated using either overly simplistic or overly complex cell line and animal model systems, which may not accurately reflect the effect of plant‐derived compounds on ovarian development. Ovarian organoids in vitro have similar structures and functions as ovaries and may provide a feasible approach to screen for plant‐derived compounds that promote ovarian organoid development.

Here, plant‐derived compounds were screened using ovarian organoids derived from feeder‐free FGSCs. Tetrahydroxy stilbene glucoside (TSG), Notopterol, and Puerarin were found to promote the development of ovarian organoids, with TSG demonstrating the most significant effect. Spatial transcriptome sequencing (ST‐seq) and single nucleus transcriptome sequencing (snRNA‐seq) were employed to delineate spatiotemporal atlases and transcriptomic profiles of ovarian organoids under the influence of TSG. Moreover, this study revealed that TSG promoted ovarian organoid development through the vascular endothelial growth factor A (Vegfa) – Eph receptor B2 (Ephb2) ligand‐receptor pair between granulosa cells (GCs) and oocytes. This analysis significantly enhances our understanding of female reproductive mechanisms, providing a robust technical platform for diagnosing and treating infertility. Furthermore, it establishes a foundational framework for the clinical utilization of plant‐derived compounds.

## 2 Results

### 2.1 Establishment of Ovarian Organoids Derived from Feeder‐Free Female Germline Stem Cells

**Figure 1:**
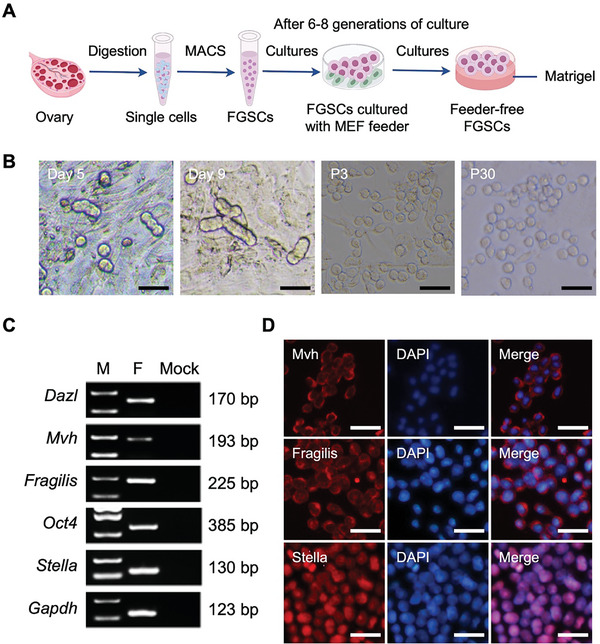
Establishment of a long‐term culture system for feeder‐free female germline stem cells (FGSCs). A. A schematic illustrating the establishment of feeder‐free FGSCs. MACS: magnetic‐activated cell sorting. B. Representative views of FGSCs cultured on day 5 (Day 5), day 9 (Day 9), and up to the 3rd generation (P3) with a feeder layer, as well as up to the 30th generation (P30) without a feeder layer under brightfield microscopy. Scale bars: 25 µm. C. RT‐PCR measurement of Dazl, Mvh, Fragilis, Oct4, and Stella mRNA expression in feeder‐free FGSCs. Lane M: 250 bp DNA markers, Lane F: FGSCs, Lane Mock: ddH2O. D. Immunofluorescence analysis of germ cell markers (Mvh, Fragilis, and Stella) in feeder‐free FGSCs. Nucleus were stained with DAPI. Scale bars: 25 µm.

To ensure the precise development of ovarian organoids without interference from feeder cells, we established a long‐term feeder‐free culture system for mouse FGSCs (**Figure** **1**A). Mouse FGSCs were isolated and purified as reported previously.^[^ ^19^, ^20^ ^]^ Freshly isolated FGSCs from the ovaries of 3‐5‐day‐old postnatal ICR mice were cultured onto a murine embryonic fibroblast (MEF) feeder layer. During the culture of FGSCs, grape‐like cell clusters were observed at an early stage, and FGSCs were passaged at a 1:1 ratio every 7–14 days (Figure 1B). After 3–4 passages, FGSCs exhibited enhanced proliferation with a passage ratio of 1:3‐1:4. Subsequently, FGSCs were expanded in feeder‐free cultured conditions. Upon reaching ≈80% confluence, the MEF feeder layer was removed, and FGSCs were cultured on Matrigel‐coated plates. Under these conditions, feeder‐free FGSCs displayed a morphology similarity to those on MEF feeder layers and could be passaged more than 30 times (Figure 1B). To characterize these feeder‐free FGSCs cultures, the expression of germ cell marker genes, including *Dazl*, *Mvh*, *Fragilis*, *Oct4*, and *Stella*, was examined using RT‐PCR (Figure 1C). Immunofluorescence staining further demonstrated the expression of Mvh, Fragilis, and Stella in feeder‐free FGSCs (Figure 1D). These results demonstrated the successful establishment of a long‐term culture system for FGSCs without a feeder layer.

**Figure 2:**
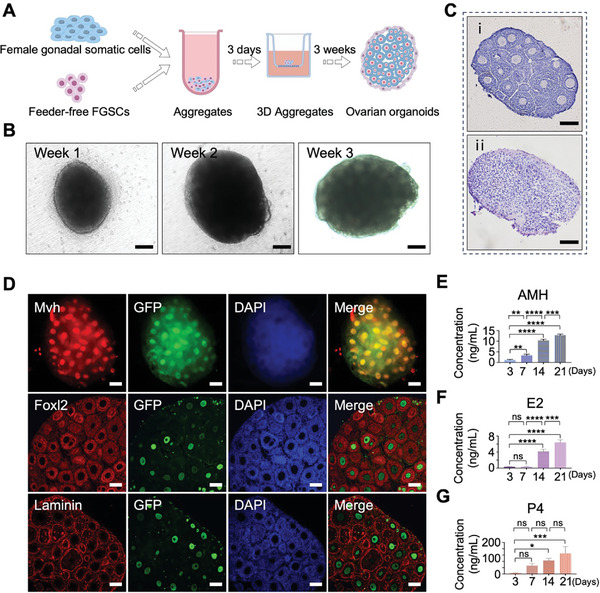
Construction of ovarian organoids derived from feeder‐free FGSCs. A. A schematic detailing the construction of feeder‐free FGSCs‐derived ovarian organoids. B. Morphology of representative ovarian organoids cultured for 1 week (week 1), 2 weeks (week 2), and 3 weeks (week 3). Scale bars: 100 µm. C. i: Hematoxylin staining of ovarian organoids cultured for 2–3 weeks. ii: Hematoxylin staining of an aggregation of mouse female gonadal somatic cells cultured for 2–3 weeks (without feeder‐free FGSCs). Scale bars: 100 µm. D. Immunofluorescence analysis for germ cells (Mvh), granulosa cells (GCs) (Foxl2), and membrane of follicle (Laminin) markers in ovarian organoids. The upper images show the expression of Mvh (red) co‐stained with GFP (green). The meddle images show the expression of Foxl2 (red) co‐stained with GFP (green). The lower images show the expression of Laminin (red) co‐stained with GFP (green). Cells were counterstained with DAPI (blue). Scale bars: 100 µm. E–G. The concentration of Anti‐Mullerian hormone (AMH) (E), estradiol (E2) (F), and progesterone (P4) (G) in the culture medium of ovarian organoids on the 3rd, 7th, 14th, and 21st days of cultivation. ^****^ *p* < 0.0001 versus Day 3; ^***^ *p* < 0.001 versus Day 3; ^**^ *p* < 0.01 versus Day 3; ns: *p* > 0.05 versus Day 3.

To generate ovarian organoids using feeder‐free FGSCs, the feeder‐free FGSCs were aggregated with female gonadal somatic cells from albino ICR mice in low‐binding U‐bottom wells (**Figure** **2**A; Figure S1, Supporting Information). As previously reported,^[^ ^9^ ^]^ aggregates formed within 3 days of culture using a GK‐15 RA medium. After this initial stage, the 3D co‐cultures were transferred to the Transwell‐COL plate membrane and cultured for another 2–3 weeks in a StemPro medium. The resulting 3D co‐cultures, formed with feeder‐free FGSCs and female gonadal somatic cells, exhibited follicle‐like structures at various developmental stages after 2–3 weeks of culture (Figure 2B,Ci). In contrast, 3D co‐cultures composed solely of female gonadal somatic cells without feeder‐free FGSCs did not display follicle‐like structures (Figure 2Cii). Then, to confirm the differentiation of oocytes derived from feeder‐free FGSCs, we constructed FGSCs that contain GFP reporter (Figure S2, Supporting Information). After 2–3 weeks of culture, oocytes within ovarian organoids were positive for GFP and Mvh (a germ cell marker). GCs surrounding the oocytes were positive for Foxl2 (a GC marker), and Laminin staining indicated the presence of a complete follicle basement membrane in ovarian organoids (Figure 2D). ELISA analysis revealed a sustained increase in anti‐Mullerian hormone (AMH) concentrations in the culture medium of ovarian organoids over time (Figure 2E). Additionally, the concentration of estradiol (E2) produced by ovarian organoids remained stable during the first week and increased with further culture (Figure 2F). The concentration of progesterone (P4) also gradually increased during ovarian organoid culture (Figure 2G). Together, these results demonstrated the successful generation of ovarian organoids using feeder‐free FGSCs.

### 2.2 Screening of Plant‐Derived Compounds Revealed that Tetrahydroxy Stilbene Glucoside Promoted the Development of Ovarian Organoids

**Figure 3:**
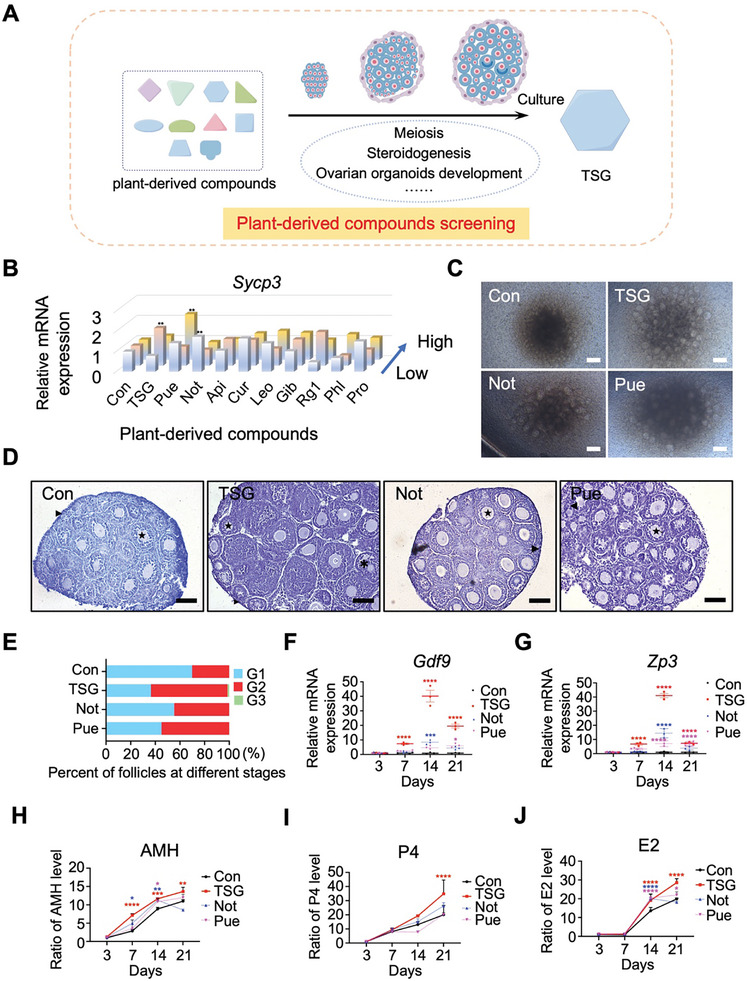
Screening and validation of plant‐derived compounds using ovarian organoids. A. The workflow for screening and validating plant‐derived compounds based on ovarian organoids. B. The expression of *Sycp3* in ovarian organoids after 3 days of treatment with gradient concentrations of plant‐derived compounds from different plants. Con: Control (DMSO/Medium); TSG: Tetrahydroxy stilbene glycoside (6.25, 25, 100 µm); Pue: Puerarin (3.125, 12.5, 50 µm); Not: Notopterol (3.125, 6.25, 12.5 µm); Api: Apigenin (3.125, 6.25, 12.5 µm); Cur: Curcumin (1.25, 2.5, 5 µm); Leo: Leonurine (20, 40, 80 µm); Gib: Gibberellin (0.05, 0.5, 5 µm); Rg1: Ginsenoside Rg1 (12.5, 25, 50 µm); Phl: Phloretin (1, 5, 25 µm); Pro: Proanthocyanins (3.125, 6.25, 12.5 µm). The arrow points in the direction of increasing concentration of plant‐derived compounds from low to high. ^**^ *p* < 0.01 versus Con. C. Morphology of ovarian organoids after 2–3 weeks of culture and treatment with TSG, Not, and Pue. D. Hematoxylin staining of ovarian organoids after 2–3 weeks of culture with treatment with TSG, Not, and Pue. Scale bars: 100 µm. ►: G1 (Primordial or primary follicles); ★: G2 (Secondary follicles); ✱: G3 (Antral follicles). E. The proportion of follicles at different developmental stages in ovarian organoids after 2–3 weeks of culture with TSG, Not, and Pue. G1: Primordial or primary follicles; G2: Secondary follicles; G3: Antral follicles. n values: the number of ovarian organdies for the statistical analyses. Con: n = 11; TSG: n = 11; Not: n = 9; Pue: n = 7. F‐G. Comparison of the mRNA expression level of germ cell markers *Gdf9* (F) and *Zp3* (G) in ovarian organoids treated with TSG, Not, and Pue on the 3rd, 7th, 14th, and 21st day of culture. ^****^ *P *< 0.0001 versus Con Day 3; ^***^ *P *< 0.001 versus Con Day 3; ^*^ *P *< 0.05 versus Con Day 3. H‐J. ELISA of the concentrations of Anti‐Mullerian hormone (AMH) (H), progesterone (P4) (I), and estradiol (E2) (J) in the culture medium of ovarian organoids treated with TSG, Not, and Pue on the 3rd, 7th, 14th, and 21st day of culture. ^****^ *P *< 0.0001 versus Con Day 3; ^***^ *P *< 0.001 versus Con Day 3; ^**^ *P *< 0.01 versus Con Day 3; ^*^ *P *< 0.05 versus Con Day 3.

To evaluate the feasibility of using ovarian organoids as screening tools for assessing the actions of factors on ovarian organoids development, 10 plant‐derived compounds (TSG,^[^ ^21^ ^]^ Notopterol,^[^ ^22^ ^]^ Puerarin,^[^ ^23^ ^]^ Apigenin,^[^ ^24^ ^]^ Curcumin,^[^ ^25^ ^]^ Procyanidine,^[^ ^26^ ^]^ Ginsenoside Rg1,^[^ ^27^ ^]^ Phloretin,^[^ ^28^ ^]^ Leonurine,^[^ ^29^ ^]^ and Gibberellins^[^ ^30^ ^]^) were selected to investigate their potential to promote ovarian organoid development (**Figure** **3**A). After 3 days of treatment with 10 plant‐derived compounds, the morphology of organoids displayed similarities with control ovarian organoids. *Sycp3*, a marker of meiosis, was expressed in the ovarian organoids from day 3 of culture, as reported previously,^[^ ^9^ ^]^ and significantly up‐regulated in ovarian organoids treated with TSG, Notopterol, and Puerarin (Figure 3B). At 2–3 weeks of culture, more secondary follicle‐like structures were observed in ovarian organoids treated with TSG, Notopterol, or Puerarin compared with the control group (Figure 3C). Hematoxylin staining also showed a notable increase in the percentage of secondary follicles‐like in ovarian organoids treated with TSG, Notophanol, and Puerarin, particularly in the TSG‐treated group (Figure 3D,E). Moreover, a few preantral‐like follicles were observed in the TSG‐treated ovarian organoids (Figure 3D,E). In addition, RT‐qPCR analysis showed that TSG‐treated ovarian organoids had the highest expression levels of germ cell markers, such as *Gdf9* and *Zp3* (Figure 3F,G). The production of steroid hormones provides a vital indicator for evaluating ovarian organoid development. TSG induced higher levels of AMH and P4 compared with the increased levels induced by Notopterol or Puerarin (Figure 3H,I). E2 production was detected after 1 week of culture, with E2 concentrations increasing in ovarian organoids treated with TSG, Notopterol, or Puerarin, with the highest level observed in the TSG‐treated group (Figure 3J). Thus, among the 10 compounds studied, TSG was particularly effective at promoting ovarian organoid development.

### 2.3 Spatial and Single Cell Transcription Landscapes of Tetrahydroxy Stilbene Glucoside‐Treated and Control Ovarian Organoids

**Figure 4:**
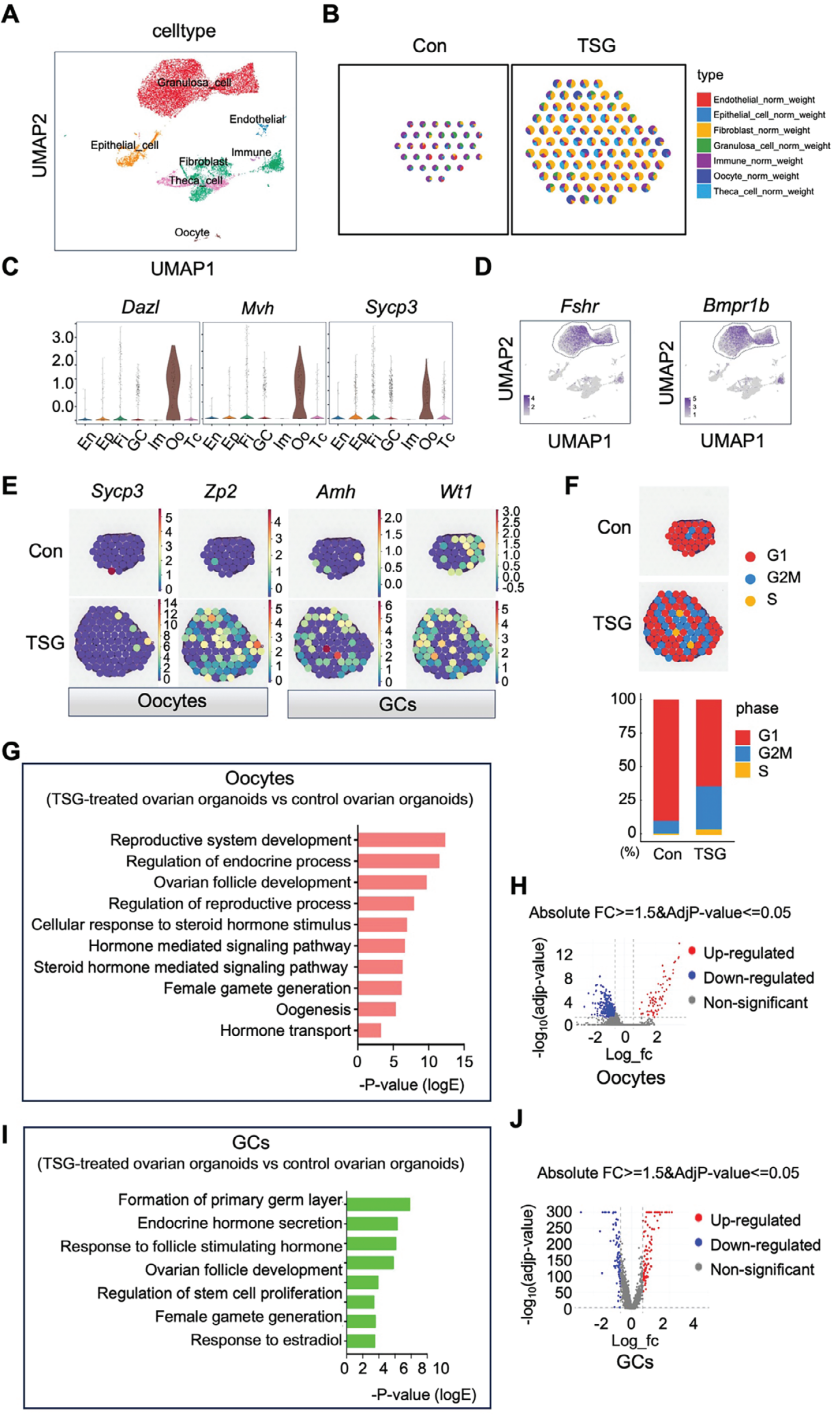
Spatial transcriptome sequencing (ST‐seq) and single nucleus transcriptome sequencing (snRNA‐seq) were employed to identify major cell types and performed Gene Ontology (GO) enrichment analysis in oocytes and granulosa cells (GCs) from tetrahydroxy stilbene glucoside (TSG)‐treated and control ovarian organoids. A. Uniform manifold approximation and projection (UMAP) visualization depicting cell types in ovarian organoids. Each dot presents an individual cell, with colors indicating different cell types. B. Cell type prediction overlay on ST‐seq spots indicating cells inferred from snRNA‐seq. C. Violin plot depicting the expression levels of germ cell markers *Dazl*, *Mvh*, and *Sycp3*. En: Endothelial; Ep: Epithelial_cell; Fi: Fibroblast; GC: Granulosa_cell; Im: Immune; Oo: Oocyte; Tc: Theca_cell. D. UMAP plot depicting the expression pattern of the GCs marker *Fshr* and *Bmpr1b*. E. Spatial plots illustrating the expression patterns of marker genes for germ cells (*Sycp3* and *Zp2*) and GCs (*Amh* and *Wt1*) in control and TSG‐treated ovarian organoids. F. ST spots and bar plots displayed the proportion of each cell‐cycle state in control and TSG‐treated ovarian organoids. G. Significantly upregulated GO term (biological processes) of differentially expressed genes (DEGs) in oocytes of TSG‐treated ovarian organoids. H. Volcano map of DEGs in oocytes between TSG‐treated and control ovarian organoids. I. Significantly upregulated GO term (biological processes) of DEGs in GCs of TSG‐treated ovarian organoids. J. Volcano map of DEGs in GCs between TSG‐treated and control ovarian organoids.

An analysis integrating ST‐seq and snRNA‐seq data from TSG‐treated and control ovarian organoids was used to explore the mechanism of TSG‐induced development of ovarian organoids. Following quality control procedures, a total of 8241 nuclei from control ovarian organoids and 6961 nuclei from TSG‐treated ovarian organoids were obtained. Low‐expressed genes and low‐quality spots were removed from ST‐seq data. Subsequently, 25 cell clusters were assigned within ovarian organoids based on the expression of recognized cell‐type‐specific markers (Figure S3, Supporting Information). Seven main cell types, including oocytes (n = 110), GCs (n = 9418), theca cells (n = 1244), immune cells (n = 42), endothelial (n = 192), epithelial cells (n = 1005), and fibroblasts (n = 3191) were annotated. The location and fractions of the above cell types in TSG‐treated and control ovarian organoids were visualized using the deep‐learning‐based method (**Figure** **4**A,B). The expression profiles of representative specific marker genes for oocytes, GCs, and theca cells were also depicted (Figure 4C,D; Figure S4A, Supporting Information). The spots of marker genes of the oocyte (*Sycp3* and *Zp2*), GC (*Amh* and *Wt1*), and theca cell (*Cyp11a1* and *F3*) were visualized (Figure 4E; Figure S4B, Supporting Information). Cell cycle analysis revealed an increased proportion of cells in the S and G2 M phases in TSG‐treated ovarian organoids compared with controls (Figure 4F). Differential gene expression analysis identified the up‐regulated and down‐regulated genes of oocytes, GCs, and theca cells, visualized through volcano plots, and Gene Ontology (GO) analysis highlighted the enriched upregulated biological processes associated with reproduction in TSG‐treated ovarian organoids for oocytes, GCs, and theca cells (Figure 4G–J; Figure S4C,D, Supporting Information). For example, oocytes exhibited activation of reproductive processes, including reproductive system development, ovarian follicle development, female gamete generation, and oogenesis. The GCs showed enrichment in biological processes related to the formation of the primary germ layer, endocrine hormone secretion, and ovarian follicle development. Furthermore, the Kyoto Encyclopedia of Genes and Genomes (KEGG) analysis identified upregulated signaling pathways related to reproduction. Interestingly, oocytes, GCs, and theca cells shared the upregulated transforming growth factor‐beta (TGF‐β) signaling pathway in TSG‐treated ovarian organoids (Figure S5A–C, Supporting Information). Analysis of differentially expressed genes (DEGs) specific to the TGF‐β signaling pathway across these three cell types identified numerous upregulated genes. For example, *Inha*, *Bmpr1b*, *Bmpr2*, *Bmp3*, and *Sptbn1* were upregulated in oocytes, *Inhbb*, *Bmp2*, *Tgfbr1*, *Tgfbr3*, *Inha*, *Bmp6*, *Smad9*, *Acvr2b*, *Acvr1*, and *Bmp3* were upregulated in GCs, and *Bmp3*, *Inha*, *Acvr1c*, *Inhba*, *Bmpr2*, *Inhbb*, *Bmpr1b*, and *Tgfbr1* were upregulated in theca cells of TSG‐treated ovarian organoids (Figure S5D–F, Supporting Information).

**Figure 5:**
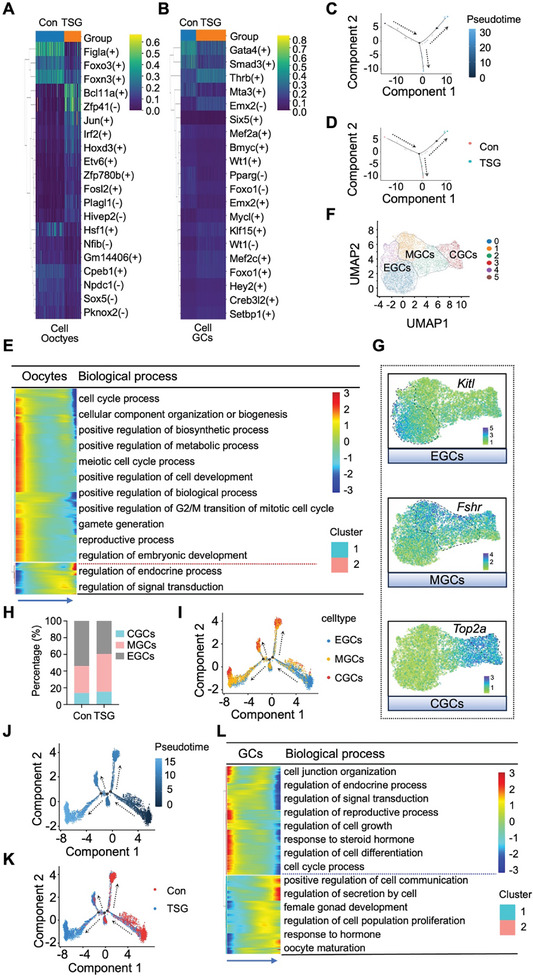
The cellular transcriptional trajectory during oocyte and granulosa cell (GC) differentiation in tetrahydroxy stilbene glucoside (TSG)‐treated ovarian organoids. A‐B. Heatmap generated by pySCENIC illustrating transcription factor (TF) activity in oocytes (A) and GCs (B) for control and TSG‐treated ovarian organoids. C. Pseudo‐time trajectory of oocytes in a 2D state space. D. Pseudo‐time trajectory of oocytes in control and TSG‐treated ovarian organoids. E. Heatmap illustrating two gene modules of significant differentially expressed genes (DEGs) and enriched Gene Ontology (GO) biological process in oocytes along the pseudo‐timeline. F. Uniform manifold approximation and projection (UMAP) reveals three subpopulations of GCs. EGCs: early granulosa cells; MGCs: mural granulosa cells; CGCs: cumulus granulosa cells. G. UMAP visualization depicting the GCs markers *Kitl*, *Fshr*, and *Top2a*. EGCs: early granulosa cells; MGCs: mural granulosa cells; CGCs: cumulus granulosa cells. H. The proportion of each developmental stage GCs (relative to the total number of GCs) in control and TSG‐treated ovarian organoids. EGCs: early granulosa cells; MGCs: mural granulosa cells; CGCs: cumulus granulosa cells. I. The pseudo‐time trajectory of GCs subpopulations along the pseudo‐time trajectory. J. Pseudo‐time trajectory of GCs. K. Pseudo‐time trajectory of GCs in control and TSG‐treated ovarian organoids. L. Heatmap illustrating two gene modules of significant DEGs and enriched GO biological process in GCs along the pseudo‐timeline.

To elucidate the transcriptional states across ovarian organoids under TSG treatment compared to control, pySCENIC was employed to identify co‐expression modules and their associated cis‐regulatory elements. This analysis indicated the increased activity of various transcription factors (TFs), such as *Bcl11a, Irf2, Hoxd3, Jun*, and *Zfp41* in oocytes, *Thrb, Setbp1, Creb3l2, Foxo1*, and *Wt1* in GCs, and *Foxp2* in theca cells in TSG‐treated versus control ovarian organoids (**Figure** **5**A,B; Figure S6A–D, Supporting Information). Conversely, pySCENIC analysis also identified TFs with reduced activity, such as *Pknox2, Npdc1, Figla, Hsf1, Sox5, Gm14406, Foxo3, Foxn3*, and *Cpeb1* in oocytes, *Bmyc, Wt1*, and *Foxo1 in* GCs, and *Zeb1 in* theca cells, in TSG‐treated ovarian organoids (Figure S6E–G, Supporting Information). Moreover, cell type‐specific TFs for oocytes, GCs, and theca cells were identified (Figure S7, Supporting Information). The transcriptional regulatory network corresponding to cell types (oocytes, GCs, and theca cells) was investigated and preferential transcriptional regulons were found in each cell type (Figures S8–S11, Supporting Information). Pseudo‐time trajectory analysis was performed on oocytes, GCs, and theca cells in ovarian organoids using Monocle2.^[^ ^31^ ^]^ Pseudo‐time analysis of oocytes revealed one branch point that diverges into two conditional specific branches (Figure 5C). The representative oocytes genes were displayed with pseudo‐time trajectories, highlighting the developmental stages (Figure S12A, Supporting Information). At the level of individual groups, cells from TSG‐treated ovarian organoids were enriched in late oocyte developmental states, contrasting with control organoids enriched in an early state, suggesting that TSG accelerated the development of germ cells in ovarian organoids (Figure 5D; Figure S12B, Supporting Information). Moreover, when analyzing the biological processes and signaling pathways of oocytes in TSG‐treated ovarian organoids based on the DEGs according to pseudo‐time trajectories, two clusters were found with different expression patterns, and these clusters were enriched with different biological processes and signaling pathways (Figure 5E). Subsequently, to perform pseudo‐time trajectory analysis on GCs, we conducted a subtype analysis of GCs first. Six subclusters of GCs were divided (Figure S13A, Supporting Information). According to the profile of marker genes of GCs, three subtypes of GCs were delineated associated with follicular development, including early granulosa cells (EGCs), mural granulosa cells (MGCs), and cumulus granulosa cells (CGCs) (Figure 5F). *Kitl*, *Fshr*, and *Top2a* were specifically highly expressed in EGCs, MGCs, and CGCs, respectively (Figure 5G). The numbers of MGCs and CGCs were significantly higher in TSG‐treated versus control ovarian organoids (Figure 5H). Immunofluorescence staining revealed a higher ratio of follicles expressing Fshr in TSG‐treated ovarian organoids than in control ovarian organoids, and a lower ratio of follicles expressed Foxl2 in TSG‐treated ovarian organoid (Figure S13B, Supporting Information). Subsequently, Monocle2 was applied to explore the pseudo‐time trajectories of GCs. Specific markers of GCs, such as *Amh, Amhr2, Foxl2, Kitl, Wnt6*, and *Wt1*, primarily expressed in the initial stage, *Fshr*, predominates in the intermediate stage, and *Top2a, Hsd17b1, Inhba*, and Inhbb mainly expressed in the late stage of developmental (Figure S13C, Supporting Information). Meanwhile, the EGCs (sub_cluster 0, 4) are predominantly located at the beginning of the developmental trajectory, followed by MGCs (sub_cluster 1, 2, and 5) in the middle stage of development, and finally CGCs (sub_cluster 3) (Figure 5I; Figure S13D, Supporting Information). This order aligned with the developmental trajectory of GCs in pseudo‐time trajectories analysis (Figure 5J). When conducting pseudo‐time trajectories analysis for each group individually, most of the TSG‐treated GCs were found to be at the late stage of the developmental trajectory, while the control group was predominantly at the early stage (Figure 5K; Figure S13E,F, Supporting Information). In addition, to evaluate the transcriptional program governing the development of GCs under the influence of TSG, we identified two clusters exhibiting distinct expression patterns along the developmental trajectory and found that these two gene clusters were enriched for different biological processes. These results revealed that TSG promoted the development of GCs in ovarian organoids (Figure 5L). Pseudo‐time trajectories of theca cells revealed alterations in several vital genes along the temporal development trajectory (Figure S14A,B, Supporting Information). Marker genes of theca cells (*Cyp11a1* and *Cyp17a1*) were elevated in TSG‐treated ovarian organoids (Figure S14C, Supporting Information). In addition, two clusters with distinct expression patterns were identified along the development trajectory, revealing the transcriptional characteristics underlying the development of theca cells under the influence of TSG (Figure S14D, Supporting Information). Taken together, transcriptional trajectory analysis revealed that TSG accelerated the development of ovarian organoids.

### 2.4 The Vegfa‐Ephb2 Ligand‐Receptor Pair Between Granulosa Cells and Oocytes is Critical in Tetrahydroxy Stilbene Glucoside‐Mediated Ovarian Organoid Development

**Figure 6:**
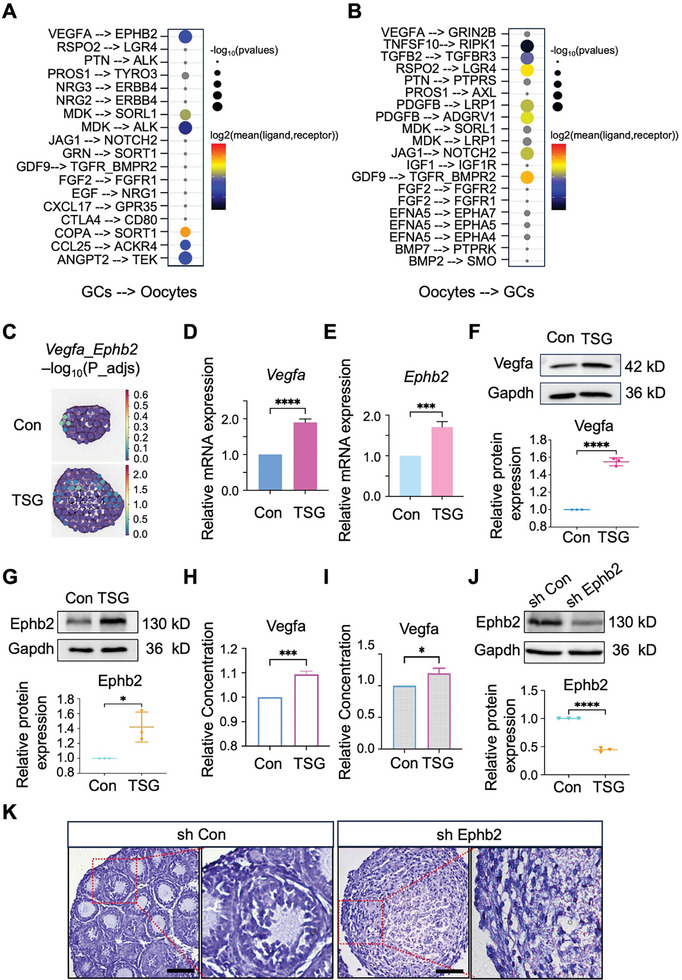
The Vegfa‐Ephb2 ligand‐receptor pair between granulosa cells (GCs) and oocytes is critical in tetrahydroxy stilbene glucoside (TSG)‐mediated promotion of ovarian organoid development. A and B. Dotted heatmap depicting the expression and significance of ligand‐receptor pairs between oocytes and GCs in ovarian organoids treated with TSG. The left side of the horizontal line represents the cell housing the signal sender, while the right side represents the cell housing the signal receiver. C. ST spots indicating the significance of the Vegfa‐Ephb2 pair in control and TSG‐treated ovarian organoids. D and E. Comparison of the mRNA expression level of *Vegfa* (D) and *Ephb2* (E) in ovarian organoids treated with TSG. ^****^ *p* < 0.0001 versus Con; ^***^ *p* < 0.001 versus Con. F and G. Detection of Vegfa (F) and Ephb2 (G) proteins in control and TSG‐treated ovarian organoids by western blot. ^****^ *p* < 0.0001; ^*^ *p* < 0.05. H and I. ELISA detected Vegfa levels in the culture medium of ovarian organoid (H) and primary GCs (I) treated with TSG. ^***^ *p* < 0.001; ^*^ *p* < 0.05. J. Detection of Ephb2 proteins in control and Ephb2‐knock down (KD) feeder‐free female germline stem cells (FGSCs) by western blot. ^****^ *p* < 0.0001. K. Hematoxylin staining of ovarian organoids. sh Con: ovarian organoids derived from control feeder‐free FGSCs; sh Ephb2: ovarian organoids derived from Ephb2‐KD feeder‐free FGSCs. The image on the right is a magnified view of the area highlighted in the red frame on the left.

To explore the communication between oocytes and GCs, the ligand‐receptor (LR) pairs between oocytes and GCs within ovarian organoids were characterized. As expected, diverse and intricate patterns of LR pair interactions between oocytes and GCs were displayed. Several high interaction score LR pairs involved receptors on oocytes and ligands produced by GCs, including Vegfa‐Ephb2, Mdk‐Alk, Mdk‐Sorl1, Copa‐Sort1, Ccl25‐Ackr4, and Angpt2‐Tek, were enriched in TSG‐treated ovarian organoids (**Figure** **6**A). Additionally, LR pairs of receptors on GCs and ligands generated by oocytes were found in ovarian organoids, such as Tgfb2‐Tgfbr3, Jag1‐Notch2, and Gdf9‐Tgfr/Bmpr2 (Figure 6B).

Based on communication analysis of oocytes regulated by GCs, three LR pairs (Vegfa‐Ephb2, Mdk‐Alk, and Angpt2‐Tek) showed significant differences between TSG‐treated ovarian organoids and the control group, with *Vegfa* exhibiting upregulated expression (Figure S15A, Supporting Information). Thus, these results indicated that the Vegfa‐Ephb2 pair played a crucial role in ovarian development under TSG treatment. To further validate their interaction, the expression of the *Vegfa*‐*Ephb2* pair was mapped onto ST‐seq and compared their co‐expression in GCs and oocytes of ovarian organoids between the TSG‐treated and control groups. The results showed that the *Vegfa*‐*Ephb2* pair was co‐expressed in ovarian organoids and increased in the TSG‐treated group, indicating that the spatial interaction of *Vegfa*‐*Ephb2* pair in TSG‐treated ovarian organoids was higher than in control ovarian organoids (Figure 6C). Immunofluorescent staining to determine the localization of the Vegfa and Ephb2 in ovarian organoids showed that both Vegfa and Ephb2 were located on the oocyte membrane of ovarian organoids (Figure S15B, Supporting Information). Subsequently, RT‐qPCR showed increased *Vegfa* and *Ephb2* expression in TSG‐treated ovarian organoids (Figure 6D,E), and western blotting demonstrated elevated levels of Vegfa and Ephb2 protein in ovarian organoids treated with TSG (Figure 6F,G). Next, ELISA was performed to assess whether TSG promoted Vegfa production by GCs in ovarian organoids. ELISA showed that the Vegfa content was higher in the culture medium of TSG‐treated ovarian organoids compared with the control (Figure 6H). Furthermore, Vegfa content in the culture medium of primary GCs isolated from 3‐week‐old ICR female mice was also increased after treatment with TSG (Figure 6I), suggesting that TSG enhanced Vegfa production by GCs. Subsequently, to investigate the role of Ephb2, predicted to be a receptor of Vegfa in oocytes, in the development of germ cells within ovarian organoids, Ephb2‐knockdown (Ephb2‐KD) feeder‐free FGSCs were generated via lentivirus‐mediated transfection (Figure 6J). Dysplastic follicles were observed in ovarian organoids generated by 3D co‐culturing Ephb2‐KD feeder‐free FGSCs with female gonadal somatic cells, highlighting the significant role of Ephb2 in ovarian organoids development (Figure 6K). To investigate whether the abnormal biological function of FGSCs within ovarian organoids was due to Ephb2 deficiency, we analyzed bulk RNA‐seq data in Ephb2‐KD FGSCs compared with controls. GO enrichment revealed that *Ephb2* was associated with biological processes such as steroid metabolic process and response to steroid hormone (Figure S16A, Supporting Information). Additionally, KEGG analysis demonstrated signaling pathways involving the Ephb2 receptor, including the HIF‐1 signaling pathway and MAPK signaling pathway (Figure S16B, Supporting Information).

## 3 Discussion

Emerging evidence suggested that plant‐derived compounds possessed multiple beneficial effects on female reproduction.^[^ ^13^, ^14^, ^15^, ^16^, ^17^, ^18^ ^]^ However, in these previous studies, the most commonly used experimental models were either simple cell lines or complex animal models, indicating the challenge in researching the mechanisms of plant‐derived compounds on female reproduction development. In recent years, organoid technology has become an emerging experimental model in various research fields.^[^ ^9^, ^32^, ^33^ ^]^ In our previous study, ovarian organoids derived from FGSCs have been successfully constructed.^[^ ^9^ ^]^ However, these FGSCs were cultured on a feeder layer, potentially leading to the inclusion of feeder layer cells in the ovarian organoids. In the current study, plant‐derived compounds were screened using our newly constructed ovarian organoids derived from feeder‐free FGSCs, revealing that TSG extracted from *Polygonum multiflorum* promoted ovarian organoid development.

Single‐cell (single nucleus) RNA transcriptome sequencing (scRNA‐seq/snRNA‐seq), the state‐of‐the‐art technology for elucidating the heterogeneity and complexity of transcription profiles within individuals, has been widely applied to annotate the composition and functions of cell types in tissues or organs.^[^ ^34^ ^]^ ST‐seq has also emerged as an advanced approach for retaining spatially resolved transcriptional information.^[^ ^35^, ^36^ ^]^ In the present study, seven cell types were identified through snRNA‐seq, and their distribution in the ovarian organoids was localized using ST‐seq. GO and KEGG annotation revealed that upregulated biological processes and signaling pathways associated with reproduction were enriched in TSG‐treated ovarian organoids, suggesting that TSG promoted ovarian organoid development. Meanwhile, pseudo‐time analysis further defined the potential actions of TSG on the development of ovarian organoids. Subsequently, the activities of TFs, such as *Jun*, *Bcl11a*, *Hoxd3*, *Zfp41*, and *Irf2* in oocytes, and *Setbp1*, *Creb3l2*, *Thrb*, *Wt1*, and *Foxo1* in GCs, were found to be increased in TSG‐treated ovarian organoids. Considering the transcriptional regulatory network of oocytes, *Bcl11a*, is vital for the function and communication of oocytes and GCs,^[^ ^37^ ^]^ and *Jun*, which is associated with primordial follicle activation,^[^ ^38^ ^]^ was found to function in the top regulons of TSG‐treated versus control ovarian organoids (Figures S8 and S11A,B, Supporting Information). *Ptprd*, which has been reported to be important for the normal progress in reproduction,^[^ ^39^, ^40^, ^41^ ^]^ served as the shared target gene of both *Bcl11a* and *Jun*, and was also increasingly expressed in oocytes in TSG‐treated ovarian organoids, indicating that *Ptprd* played a significant role in the TSG‐induced development of ovarian organoids (Figure S11C, Supporting Information). For the transcriptional regulatory network of GCs, the activity of *Thrb* and *Wt1* was prominently increased in TSG‐treated ovarian organoids. *Thrb*, a candidate gene for ovarian development,^[^ ^42^ ^]^ was found to have an activation effect on its target genes. *Wt1*, essential for the differentiation of GCs,^[^ ^43^ ^]^ primarily exhibited a repressing influence on its target genes. Notably, *Zfp385b*, a shared target gene for *Wt1* and *Emx2*, was also upregulated in TSG‐treated ovarian organoids (Figure S11D, Supporting Information).

Cell‐cell communication via LR pairs is vital in physiological and pathological processes.^[^ ^44^ ^]^ Based on snRNA‐seq and ST‐seq analysis, the current study captured LR pairs between GCs and oocytes in ovarian organoids and revealed a higher communication score of the Vegfa‐Ephb2 pair compared with other LR pairs. The expression level of *Vegfa* and *Ephb2*, as well as the secretion of Vegfa, were increased in TSG‐treated ovarian organoids. Vegfa has been linked to degeneration of follicle reserve, follicular development, atresia, and GCs apoptosis,^[^ ^45^, ^46^, ^47^, ^48^, ^49^ ^]^ suggesting Vegfa has key functions in ovarian development. Eph receptor tyrosine kinases interact with their ligands and are involved in the development of germ layers during gastrulation.^[^ ^50^, ^51^ ^]^ However, how Eph receptors regulate ovarian development remains poorly understood. In the present study, *Ephb2* deficiency in ovarian organoids resulted in dysplastic follicles. Moreover, bulk RNA‐seq and Ephb2‐KD analysis indicated that Ephb2 has an important role in reproductive processes. These findings demonstrated that disruption of *Ephb2* may inhibit ovarian organoid development. In support of this, a previous study reported that *Ephb2* was involved in the formation of the corpus luteum.^[^ ^52^ ^]^ Thus, we predicted that Vegfa‐Ephb2 interaction plays a crucial role in the development of ovarian organoids or ovaries. Notably, Eph receptors have been reported to be one of the largest known subfamily of receptor protein‐tyrosine kinases.^[^ ^53^ ^]^ Based on the ligand‐binding specificity, Eph receptors are further divided into Epha and Ephb receptors,^[^ ^54^ ^]^ and Ephb receptors are subdivided into six classes (Ephb1‐6).^[^ ^55^ ^]^ For Ephb2, a previous study showed that Ephrin‐A5, rather than Vegfa, was the specific ligand directing its activity.^[^ ^56^ ^]^ For Vegfa, Vegfr has been identified as the key receptor for Vegfa.^[^ ^57^ ^]^ Therefore, the interaction between Vegfa and Ephb2 may not be a direct effect. The cell communication analysis of this study indicated that TSG regulated the interaction between GCs and oocytes through the Vegfa‐Ephb2 pair in ovarian organoids, suggesting a possible synergistic effect of VEGF signaling and EPH signaling pathways exists in promoting ovarian follicular development.

Although this work depicted the transcriptional landscape of gene expression in ovarian organoids and uncovered the potential mechanism by which TSG accelerates their development, there were also some limitations. For instance, because of technical limitations, the number of oocytes obtained in this study was relatively small. In the future, advances in sequencing technology may allow the use of a large number of oocytes, facilitating more accurate exploration of the timing of oocyte development. Additionally, several crucial predictions, such as the functions of key TFs, require further validation. Moreover, the synergistic effect of VEGF signaling and EPH signaling was not investigated in this study and should be further analyzed in future studies.

Overall, we proposed a model in which TSG promoted ovarian organoids development by stimulating ovarian follicle growth and steroidogenesis through the mechanism of facilitating the Vegfa‐Ephb2 interaction between GCs and oocytes. Our findings identified the stimulatory effect of TSG on ovarian organoid development, highlighting its potential for the clinical treatment of ovarian hypofunction. This discovery opens new avenues for improving ovarian development through targeting VEGFA‐EPHB2 pair interactions.

## 4 Experimental Section

### Animals

ICR strain wild‐type female mice (embryonic day 12.5‐15.5, 3–5 days old, and 6–8 weeks old) and ICR strain wild‐type male mice (6–8 weeks old) were purchased from SLAC Laboratory (Shanghai, China). This study was performed according to recommendations in the Guide for the Care and Use of Laboratory Animals and relevant Chinese laws and regulations, approved by the Institutional Animal Care and Use Committee (IACUC) of Shanghai Jiao Tong University (Mechanisms of female germline stem cell development and ovarian function remodeling, 201 703 004).

### Feeder‐Free Expansion of Female Germline Stem Cells

Ovaries were obtained from 3‐5‐day‐old ICR wild‐type female mice. Then, the ovaries were washed with phosphate‐buffered saline (PBS) and dissected into pieces. Two‐step enzymatic methods were employed to dissociate ovary tissue into single cells.^[^ ^20^, ^58^ ^]^ To obtain purified FGSCs, cells were isolated using magnetic‐activated cell sorting (MACS) using anti‐Mvh (1:100, ab270534, abcam) and anti‐Fragilis (1:100, ab288563, abcam) antibodies, and then the Mvh‐ and Fragilis‐ positive cells were cultured on a MEF feeder layer for 6–8 generations. To establish a long‐term culture system for FGSCs without a feeder layer, plates were coated with Matrigel (356 231, Corning, diluted at a ratio of 1:10 in culture medium, added to the plates, and incubated at 37 °C with 5% CO_2_ for 30 min). Subsequently, when the cell density reached 70%–80%, FGSCs were seeded onto Matrigel‐coated plates for further culture. Purified FGSCs without feeder cells (feeder‐free FGSCs) were obtained after culturing more than two passages. Feeder‐free FGSCs were passaged at a ratio of 1:3 to 1:4 every 2–3 days and were cultured for up to 30 generations. The composition of the culture medium for FGSCs was based on previous studies.^[^ ^20^, ^58^, ^59^ ^]^

### Isolate and Culture Condition of Female Fetal Gonadal Somatic Cells

The gonads of female fetal mice were harvested from pregnant ICR wild‐type mice (embryonic day 12.5‐15.5). Then, the gonads were washed with PBS and dissociated into single cells using 1 mg mL^−1^ collagenase at 37 °C for 15–20 min with agitation. Cell pellets obtained after centrifugation were washed once with cold PBS. To remove germ cells from the gonadal cell suspension, cells were incubated with anti‐SSEA1 MicroBeads (130‐094‐530, Miltenyi Biotec) and CD31 MicroBeads (130‐097‐418, Miltenyi Biotec) for 30 min, and then isolated using MACS. Subsequently, Ssea1‐ and Cd31‐negative cells, representing purified somatic cells from female fetal mice gonads, were collected and cultured for up to 2 days before use to construct ovarian organoids. The culture medium was DMEM medium (12 100 046, gibco) supplemented with 10% fetal bovine serum (FBS) (10 099, gibco), 1× non‐essential amino acid (NEAA) (11 140 050, gibco), 1× mycillin (15 070 063, gibco).

### Construction and Culture of Ovarian Organoids

To construct ovarian organoids, feeder‐free FGSCs were aggregated with purified female gonadal somatic cells at a ratio of 1:100 in a low‐binding U‐bottom 96‐well plate (174 925, Thermo Fisher Scientific) and cultured in GK‐15 differentiation medium^[^ ^9^ ^]^ for 3 days. After 3 days of culture, the aggregated ovarian organoids were transferred onto Transwell‐COL plate (CLS3412, or CLS3421, Corning) and maintained in Stempro differentiation medium^[^ ^9^ ^]^ (without ICI182780 in this study) for 2–3 weeks. Simultaneously, female gonadal somatic cells were aggregated to serve as the negative control. The aggregated ovarian organoids were cultured at 37 °C with 5% CO_2_, with the culture medium changed every 2 days by replacing half of the volume.

### Administration of Plant‐Derived Compounds to Ovarian Organoids

Ovarian organoids were treated with 10 plant‐derived compounds: TSG (ST8110, Solarbio), Notopterol (N418583, Aladdi), Puerari (P905, Solarbio), Apigenin (62030C, Adamas), Curcumin (PHR2209, Supelco), Procyanidine (102924B, Adamas), Ginsenoside Rg1 (62030C, Adamas), Phloretin (P7912, Sigma), Leonurine (L135393, Aladdin), Gibberellins (G764, Sigma). Plant‐derived compounds were stored according to manufacturer instructions to prevent freezing and thawing. During the initial 3 days of organoid culture (GK‐15 differentiation medium), ovarian organoid were treated with vehicle (culture medium/DMSO), TSG (6.25, 25, 100 µm), Notopterol (3.125, 6.25, 12.5 µm), Pueraria (3.125, 12.5, 50 µm), Apigenin (3.125, 6.25, 12.5 µm), Curcumin (1.25, 2.5, 5 µm), Procyanidine (3.125, 6.25, 12.5 µm), Ginsenoside Rg1 (12.5, 25, 50 µm), Phloretin (1, 5, 25 µm), Leonurine (20, 40, 80 µm), and Gibberellins (0.05, 0.5, 5 µm). The concentrations of plant‐derived compounds treating ovarian organoids were determined based on integrating literature reports, instruction, and cytotoxicity assay results (not presented here). Subsequently, *Sycp3* expression was used to identify plant‐derived compounds promoting germ cell meiosis. Plant‐derived compounds that increased *Sycp3* expression were further studied by adding them to ovarian organoids culture for an additional 2–3 weeks. Evaluation of follicle development using hematoxylin staining and steroidogenesis detected by ELISA was employed to determine the most effective compound for subsequent mechanistic research.

### Histological and Morphological Analysis of Ovarian Organoids

Ovarian organoids were fixed in 4% paraformaldehyde at 4 °C for over 24 h. Subsequently, fixed ovarian organoids underwent two 40‐min washed in 1 × PBS, followed by dehydrated in a graded ethanol series (50%, 70%, 80%, 90%, 95%, 100% ethanol, 50% ethanol + 50% xylene, and 100% xylene), and were then embedded in paraffin for 4 h. The ovarian organoids were then sectioned to a thickness of 6 µm. Next, sections were subjected to two 20‐min xylene infiltrations, followed by graded rehydrated (100%, 95%, 90%, 80%, 70%, 50% ethanol, and distilled water) for 5 min each. Morphological characteristics of the ovarian organoids were visualized using hematoxylin staining, and images were captured with an optical microscope (Leica, Germany).

### Immunofluorescence Staining

For immunofluorescence staining of FGSCs, FGSCs were cultured in 48‐well plates until reaching 85% confluence and fixed with 4% paraformaldehyde at 37 °C for 30 min. Subsequently, FGSCs were blocked with 10% goat serum at 37 °C for 30 min, followed by overnight incubation with primary antibodies diluted in 1 × PBS (anti‐Mvh, 1:100, ab270534, abcam; anti‐Fragillis, 1:100, ab288563, abcam; anti‐Stella, 1:100, ab19878, abcam) at 4 °C. FGSCs were then incubated with fluorescent secondary antibodies (1:500, #8889 or #8890, CST,) at 37 °C for 1 h and stained with 500 ng mL^−1^ DAPI at room temperature for 3 min. Images were acquired by fluorescence microscope (Leica, Germany).

For immunofluorescence staining of ovarian organoids, the ovarian organoids embedded in paraffin were sectioned at 6 µm thickness and subjected to the infiltration and rehydration protocol as previously described (“*Histological and morphological analysis of ovarian organoids*” *of this paper*). Then, antigen retrieval was performed, followed by blocking in 10% goat serum. The sections were then incubated overnight at 4 °C with primary antibodies (anti‐Mvh, 1:100, ab270534, abcam; anti‐Gfp, 1:100, 55 494, CST; anti‐Foxl2, 1:100, ab246511, abcam; anti‐Laminin, 1:100, ab11575, abcam; anti‐Vegfa, 1:100, ab51745, abcam; anti‐Ephb2, 1:100, ab307811, abcam). After washing, sections were incubated with secondary antibodies (1:200, #8889, or #4408, CST) and stained with DAPI. Images were captured by fluorescence microscope (Leica, Germany).

### RT‐PCR and RT‐qPCR

Total RNA from ovarian organoids was isolated using TRNzol reagent (DP424, TIANGEN, China) and assessed for quality using a nanodrop lite spectrophotometer (Thermo Fisher Scientific, USA). Subsequently, the reverse transcription, RT‐PCR, and RT‐qPCR processes followed protocols as described in previous reports.^[^ ^9^ ^]^ Data were analyzed by the 2^−ΔΔCt^ method. The primers are shown in Table S1 (Supporting Information).

### Western Blotting

The total proteins of ovarian organoids were extracted using RIPA lysis buffer (20101ES60, YEASEN, China) supplemented with protease inhibitor, followed by collection of the supernatants. The proteins were separated by electrophoresis and transferred onto PVDF membranes (Millipore, USA). Subsequently, the membranes were blocked with 5% non‐fat milk, and then incubated with primary antibodies (anti‐Vegfa, 1:1000, ab214424, abcam; anti‐Ephb2, 1:1000, ab252935, abcam; anti‐Gapdh, 1:10 000, 60004‐1‐Ig, Proteintech, China) at 4 °C overnight. After washing, the membranes were incubated with secondary antibodies (1:2000, SA00001‐1 or SA00001‐2, Proteintech, China) at 37 °C for 1h. Protein bands were visualized using a Gel Imager System (Tanon, 4600SF, China) and analyzed using image J software.

### ELISA

To measure hormone production in ovarian organoids, the culture media from ovarian organoids were collected on the 3rd, 7th, 14th, and 21st day of culture. The concentrations of progesterone (PP773, Beyotime Biotechnology, China), Anti‐Mullerian hormone (E‐EL‐M3015, Elabscience, China), and estradiol (PE223, Beyotime Biotechnology, China) in culture mediums were analyzed using ELISA. Concentrations were calculated using a 4‐parameter logistic curve based on standard data. To measure Vegfa secretion in ovarian organoids, the culture mediums from ovarian organoids were collected on the 14th day of culture. The concentrations of Vegfa in the mediums were analyzed using ELISA (E‐EL‐M1292, Elabscience, China). Concentrations were determined using a 4‐parameter logistic curve based on standard data.

### Ovarian Organoids Transparency

Ovarian organoids were fixed in 4% paraformaldehyde at 4 °C for more than 24 h. Subsequently, the ovarian organoids were processed and stained according to the Tissue Clearing Pro Kit instructions (7389, Bio‐techno, USA). Briefly, initially, the ovarian organoids were washed twice in 1× PBS for 40 min. Next, ovarian organoids were permeabilized using Tissue Clearing Pro Tissue Permeabilization Buffer. After additional washes with PBS, ovarian organoids were dehydrated in graded ethanol (50%, 80%, and 100%), washed (20% DMSO/ethanol, 80% ethanol, 50% ethanol, 0.2% PBS supplemented with TritonX‐100), permeabilized with Tissue Clearing Pro Permeabilization Buffer, blocked with Tissue Clearing Pro Blocking Buffer, incubated with primary antibody (anti‐Mvh, 1:100, ab270534, abcam; anti‐Gfp, 1:100, 55 494, CST) and secondary antibody (1:200, #8889 or #4408, CST), washed with Tissue Clearing Pro Washing Buffer, dehydrated in graded ethanol again (50%, 80%, and 100% ethanol), and cleared with Tissue Clearing Pro Reagent 1 and Tissue Clearing Pro Reagent 2. Finally, the transparent ovarian organoids were imaged using confocal microscopy.

### Ovarian Organoids Collection and Single‐Nuclei Suspension Preparation for snRNA‐seq

Ovarian organoids were collected and washed twice with cold 1× PBS. Excess water was blotted using absorbent paper, and the ovarian organoids were quick‐frozen with liquid nitrogen for 10 min and stored at −80 °C for up to 1 week following methods described in a previous study.^[^ ^60^ ^]^ Briefly, the frozen tissue was processed in NLB buffer containing 250 mm sucrose, 10 mm Tris‐Hcl, 3 mm MgAc_2_, 0.1% Triton X‐100, 0.1 mm EDTA, 0.2 U µL^−1^ RNase inhibitor. Sucrose concentrations were varied to isolated and purified nucleus, which were adjusted to ≈1000 nuclei µL^−1^ for snRNA‐Seq. Cell qualification ensured a minimum of 90% viable cells, with a total of 10 000 cells sequenced.

### snRNA‐seq Library Preparation, Sequencing, and Data Analysis

The preparation of snRNA‐seq libraries and subsequent sequencing followed the methods described in previous studies.^[^ ^61^ ^]^ 10 × Genomics Chromium Controller Instrument and Chromium Single Cell 3′ V3.1 Reagent Kits (10 × Genomics, Pleasanton, USA) was used to generate the snRNA‐seq libraries. The original BCL file was assembled utilizing Illumina's bcl2fastq converter, and the raw data within the FASTQ file was obtained. The original sequencing data were subjected to quality control, detection, and reference genome comparison using the Cell Ranger Analysis Pipeline (version 6.0.2) (10 × Genomics, USA) and the standard pipeline. Cells were filtered out with UMI/gene numbers smaller than the threshold (UMI ≤ 100000, 200 ≤ gene numbers ≤ 8000). Following the visual inspection of cell distribution based on the mitochondrial gene expression fraction, cells with over 30% of their gene counts attributed to mitochondrial genes were further filtered out to exclude low‐quality cells. This procedure produced a gene expression matrix comparing data from 15 202 individual nuclei, which then served as the basis for subsequent analyses. Normalized count data were obtained through library size normalization, which utilized the pp.normalize_total in Scanpy (version 1.8).^[^ ^62^ ^]^ Specifically, the gene expression data for each cell were normalized employing the global‐scaling method, entailing an initial multiplication of the total expression by a default scaling factor of 10000, followed by a log transformation using the pp.log1p function. The most variable genes were assertained by the pp.highly_variable_genes function within Scanpy, following the method outlined by Macosko et al.^[^ ^62^ ^]^ In Seurat (version 4.0), the marker genes distinctive to each cluster were identified through the utilization of the FindAllMarkers function (test.use = wilcox).^[^ ^63^ ^]^ Within a specific cluster, positive marker genes were identified by FindAllMarkers in comparison to all other cells (significant marker genes were identified by thresholds set at an adjusted *p* value < 0.05 and |fold change| > 2). Subsequently, the results from FindAllMarkers were integrated with cell‐type specific markers derived from the literature for the purpose of cell annotation. Subsequently, the R package g:Profiler2^[^ ^64^ ^]^ was employed to conduct a functional enrichment analysis on the marker genes. These genes, specific to each cluster, were linked to established functional pathways, and the hypergeometric distribution test was applied to determine if these biological processes were significantly over‐represented. This analysis encompassed pathways from GO and KEGG, etc. Cell cycle analysis was performed employing the Cell Cycle Scoring method in Seurat.^[^ ^63^ ^]^

### Cell‐cell Communications Analysis

The Ligand‐receptor interactions were predicted by CellPhone DB (v2.1). The average expression levels of ligand and receptor pairs were calculated, and genes that were expressed in over 10% of the cells were selected for subsequent analysis. Significance in mean and cell communicator (*p* < 0.05) was determined based on the interactions and the normalized cell matrices obtained through Seurat Normalization.

### Pseudo‐Time Analysis

Single‐cell Trajectory analysis was conducted using Monocle2 (http://cole‐trapnell‐lab.github.io/monocle‐release) with the DDR‐Tree algorithm. The Monocle2 CellDataSet object (cds) was conducted from the normalized count matrix. The Seurat package was utilized to identify 3000 highly variable features. Dimensionality reduction was performed using the reduceDimension function from the Monocle package, with the parameters ste as follows: the maximum number of components (max_components) was limited to 2, the normalization method (norm_method) was set to “none”, scaling was applied to the data, and a pseudo‐expression value (pseudo_expr) of 0 was utilized. After dimensionality reduction, the cells were ordered in pseudotime using the orderCells function.

### Transcription Factor Network Construction

Gene regulatory network analysis was performed using pySCENIC^[^ ^65^ ^]^ (v0.10.0) to identify transcription factors (TFs) and their target genes. The workflow commenced with the creation of a gene expression matrix containing normalized counts. candidate TF‐target relationships (regulons) were first identified using the GRNBoost2 algorithm with default parameters. The co‐expression modules were then refined using RcisTarget (v1.0.0) to identify enriched transcription factor binding motifs, utilizing the mouse motif database (mm9‐tss‐centered‐10kb‐7species.mc9nr). Only modules with significantly enriched motifs (normalized enrichment score > 3.0) were retained. The activity of each regulon was then scored in each cell using the AUCell algorithm (threshold = 0.5). For the final regulon specificity analysis, regulon activity scores were calculated using a 500‐gene threshold for the AUCell analysis. The resulting regulon activity matrix was used for downstream analysis to identify cell‐type specific regulatory networks.

### Ovarian Organoids Preparation for Spatial Transcriptomic Analysis

Ovarian organoids were rinsed in cold PBS and the water was blotted. Then, the ovarian organoids were cryopreserved in OCT, and stored at −80 °C. OCT blocks were sliced to a thickness of 10 µm.

### Spatial Transcriptomics Data Analysis

The analysis pipeline receives FASTQ files and uses the Cell Ranger analysis pipeline (version 6.0.2) to process raw data. Reference genome files (mouse: mm10) and transcriptome annotation files (mouse: GENCODE vM23/Ensembl 98) are used for sequence alignment. The unique molecular identifier (UMI) count matrix generated is further subjected using the Python package Scanpy^[^ ^62^ ^]^ (version 1.8). Normalization of library size was performed employing the pp.normalize_total function in Scanpy.^[^ ^62^ ^]^ The gene expression measurements for each spot are normalized through the global‐scaling normalization method, which involves multiplying the total expression by a scaling factor (10000), followed by log transformation using the pp.log1p function. The method described by Macosko et al.^[^ ^66^ ^]^ was used to identify variable genes across spots. The selection of the most variable genes was achieved by the pp.highly variable_genes function in Scanpy.^[^ ^62^ ^]^ Graph‐based clustering of spots was performed according to their gene expression profiles using the pp.neighbors function in Scanpy.^[^ ^62^ ^]^ The visualization of the spot was achieved employed the 2D Uniform Manifold Approximation and Projection (UMAP) algorithm with the tl.umap function in Scanpy.^[^ ^62^ ^]^ Marker genes for each cluster were identified using the FindAllMarkers function in Seural^[^ ^67^ ^]^ (test use = wilcox). Within a special cluster, positive markers were identified by comparison with all other spots using FindAllMarkers (*p* value < 0.05 and |log2 foldchange| > 0.25 were set as the threshold for significant marker genes). Subsequently, the R package ClusterProfiler^[^ ^68^ ^]^ was used to perform functional enrichment analysis of the marker genes. The marker genes for each cluster were mapped to known sources of functional pathways, and the hypergeometric distribution was used to check whether these biological processes were over‐represented.

### Statistics

All data were presented as mean ± standard deviation. Differences among the two groups were assessed using *t* test. One‐way ANOVA analysis was applied for the comparison among three groups. *p* < 0.05 was regarded as statistically significant. GraphPad prism 10.0 was used to conduct statistical analysis. The schematic diagram in this paper was primarily created using Figdraw (TPISU2a120).

## Conflict of Interest

The authors declare no conflict of interest.

## Author Contributions

C.M. and X.L. contributed equally to this work. All data and work reported in the paper, and the manuscript writing were performed by C.M. and X.L, except those stated below. J.Y performed assistance with the administration of plant‐derived compounds to ovarian organoids. G.T. assisted in uploading the sequencing data. H.B. performed assistance with the administration of plant‐derived compounds to ovarian organoids. W.L. and L.W. Performed conceptualization. X.L. also performed conceptualization and funding acquisition. J.W. performed conceptualization, funding acquisition, supervision, project administration, and manuscript revision.

## Supporting information



Supporting Information

## Data Availability

The data that support the findings of this study are available from the corresponding author upon reasonable request.
