# Supplementary material for: Spatial Transcriptome and Single Nucleus Transcriptome Sequencing Reveals Tetrahydroxy Stilbene Glucoside Promotes Ovarian Organoids Development Through the Vegfa‐Ephb2 Pair
Source: Adv Sci (Weinh). 2024 Dec 4;12(4):2410098. doi: 10.1002/advs.202410098 (PMC11775562; doi:10.1002/advs.202410098)
Supplement: Supplementary file 1 — Supporting Information [file ADVS-12-2410098-s001.docx]

Supporting Information

Spatial Transcriptome and Single Nucleus Transcriptome Sequencing Reveals Tetrahydroxy Stilbene Glucoside Promotes Ovarian Organoids Development Through the Vegfa-Ephb2 Pair

*Chunlan Mu**^#^, Xiaoyong Li^#, *^, Jiamei Yang,* *Geng G. Tian, Hepeng Bai, Wenhui Lin^*^,* *Linhui Wang^*^, Ji Wu^*^*


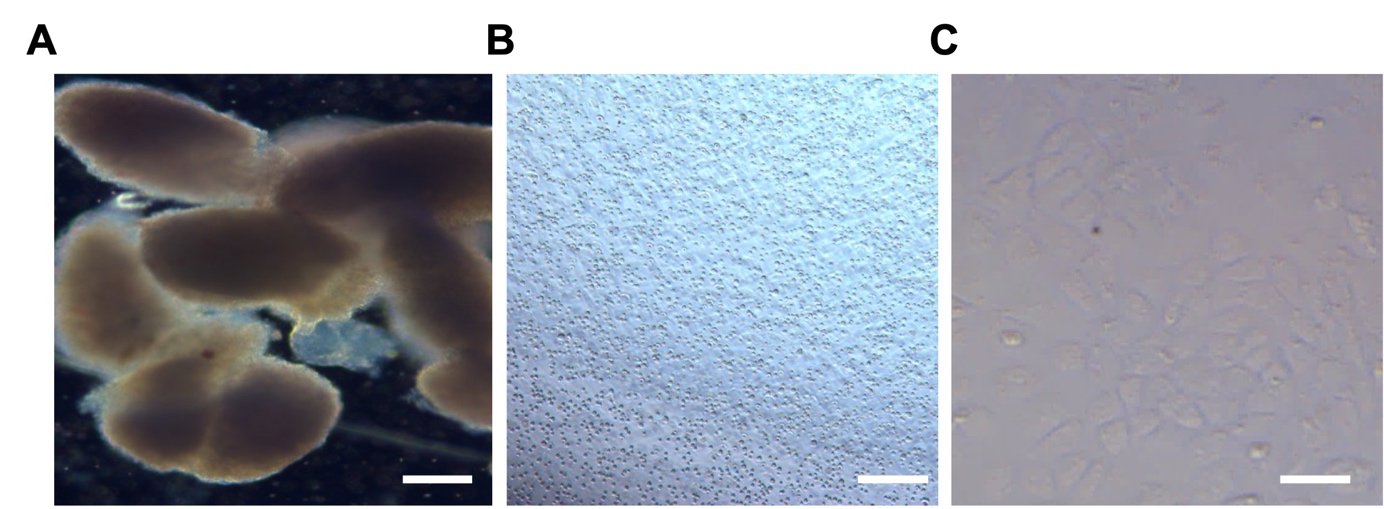


**Figure S1.** **Image of mouse female fetal gonads and female fetal mouse gonads cells captured under brightfield microscopy. A.** Image of mouse female fetal gonads (embryonic day 12.5-15.5). Scale bar: 25 μm. **B**. Image of mouse female gonadal somatic cell suspension. Scale bar: 300 μm. **C**. Image of mouse female gonadal somatic cells cultured for 2 days. Scale bar: 100 μm.


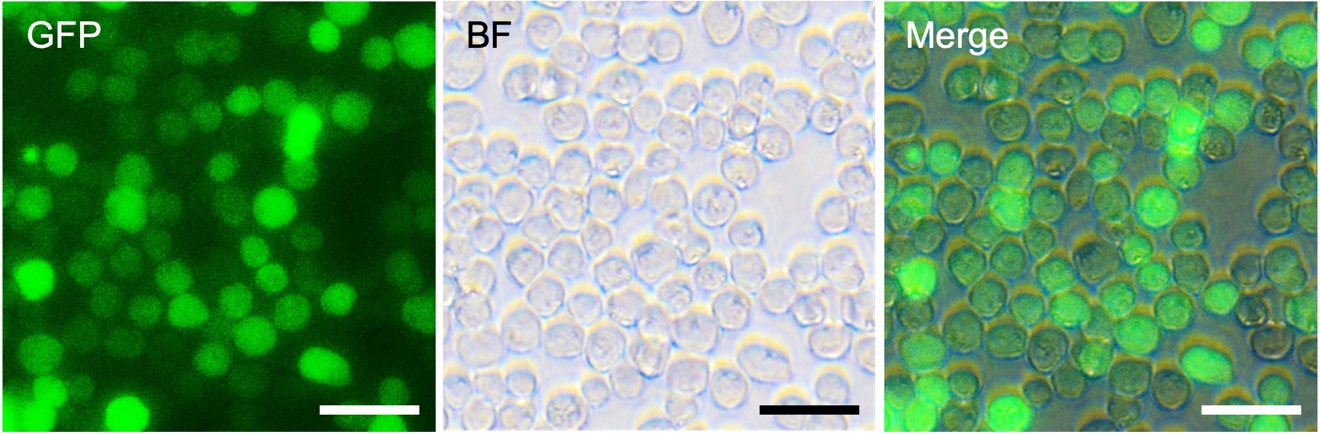


**Figure S2. Representative images feeder-free female germline stem cells (FGSCs) containing a GFP reporter.** Left: fluorescence field; meddle: brightfield; right: merged.


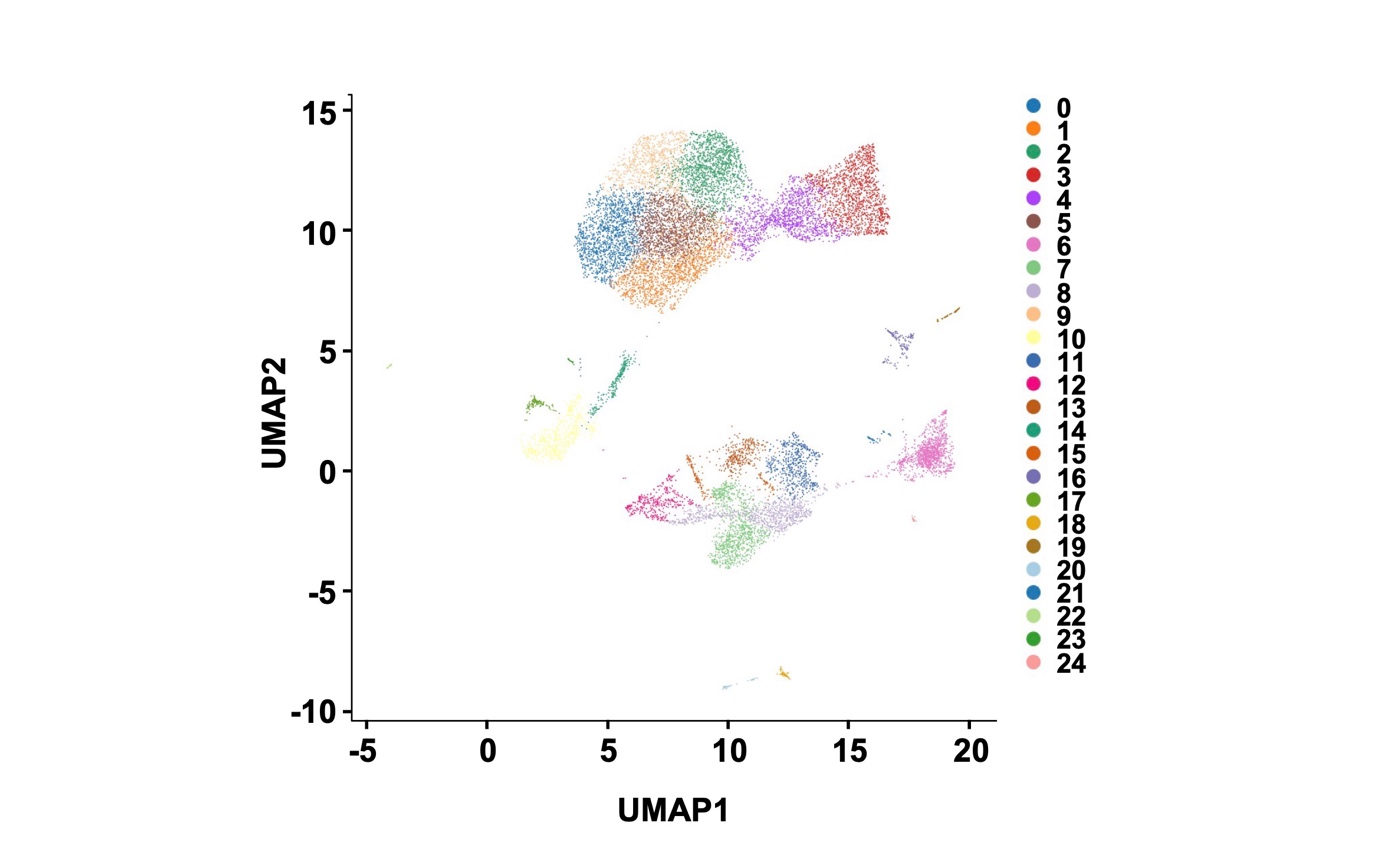


**Figure S3. Uniform manifold approximation and projection (UMAP) visualization of cells in ovarian organoids identified 25 clusters.** Each dot presents an individual cell, with colors indicating different cell clusters.


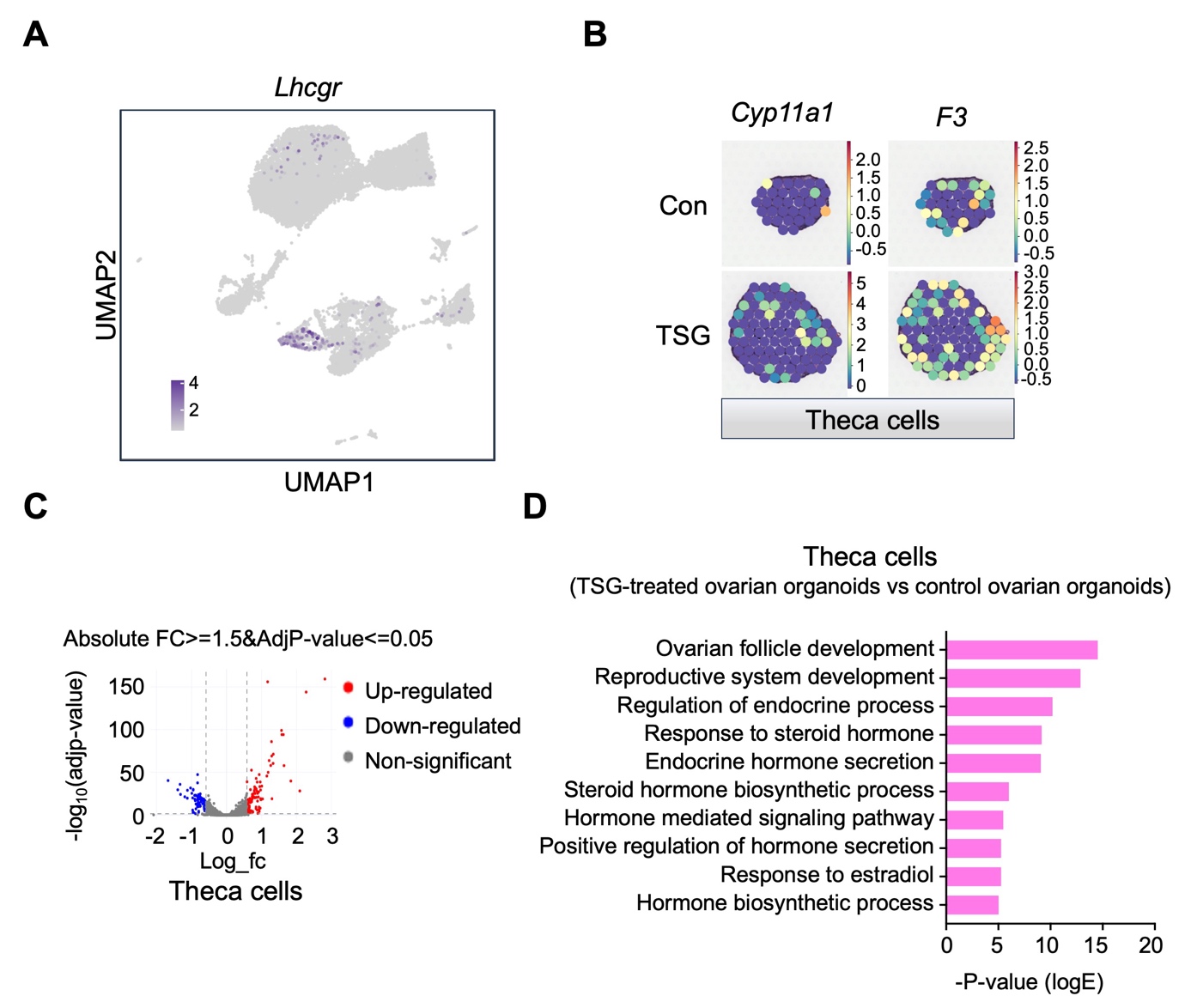


**Figure S4. Single nucleus transcriptome sequencing (snRNA-seq) and spatial transcriptome sequencing (ST-seq) were employed to identify transcripts in theca cells from control and tetrahydroxy stilbene glucoside (TSG)-treated ovarian organoids, and perform Gene Ontogeny (GO) enrichment analysis. A**. Uniform manifold approximation and projection (UMAP) showing the theca cells marker *Lhcgr*. **B**. Spatial plots illustrated the expression patterns of marker genes for theca cells (*Cyp11a1* and *F3*) in control and TSG-treated ovarian organoids. **C**. Volcano map of differentially expressed genes (DEGs) in theca cells between TSG-treated ovarian organoids and control ovarian organoids. **D**. Significantly upregulated GO term (biological processes) of DEGs in theca cells of TSG-treated ovarian organoids.


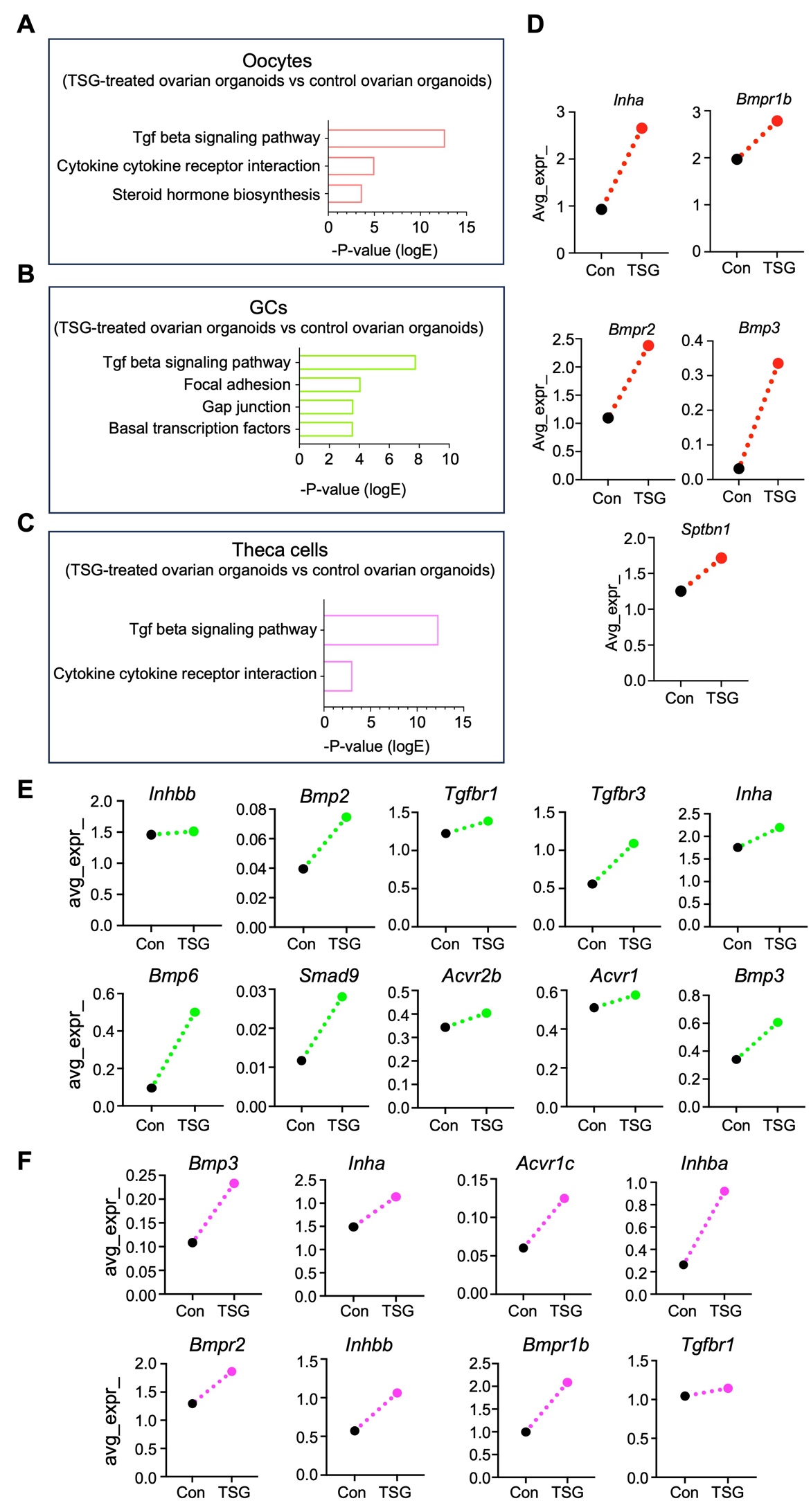


**Figure S5. Kyoto Encyclopedia of Genes and Genomes (KEGG) enrichment analysis of transcript from oocytes, granulosa cells (GCs), and theca cells from tetrahydroxy stilbene glucoside (TSG)-treated and control ovarian organoids. A-C.** Significantly upregulated signaling pathways of differentially expressed genes (DEGs) in oocytes (A), GCs (B), and theca cells (C) of TSG-treated ovarian organoids. **D-F**. Gene expression dynamics in oocytes (D), GCs (E), and theca cells (F) in TSG-treated ovarian organoids.


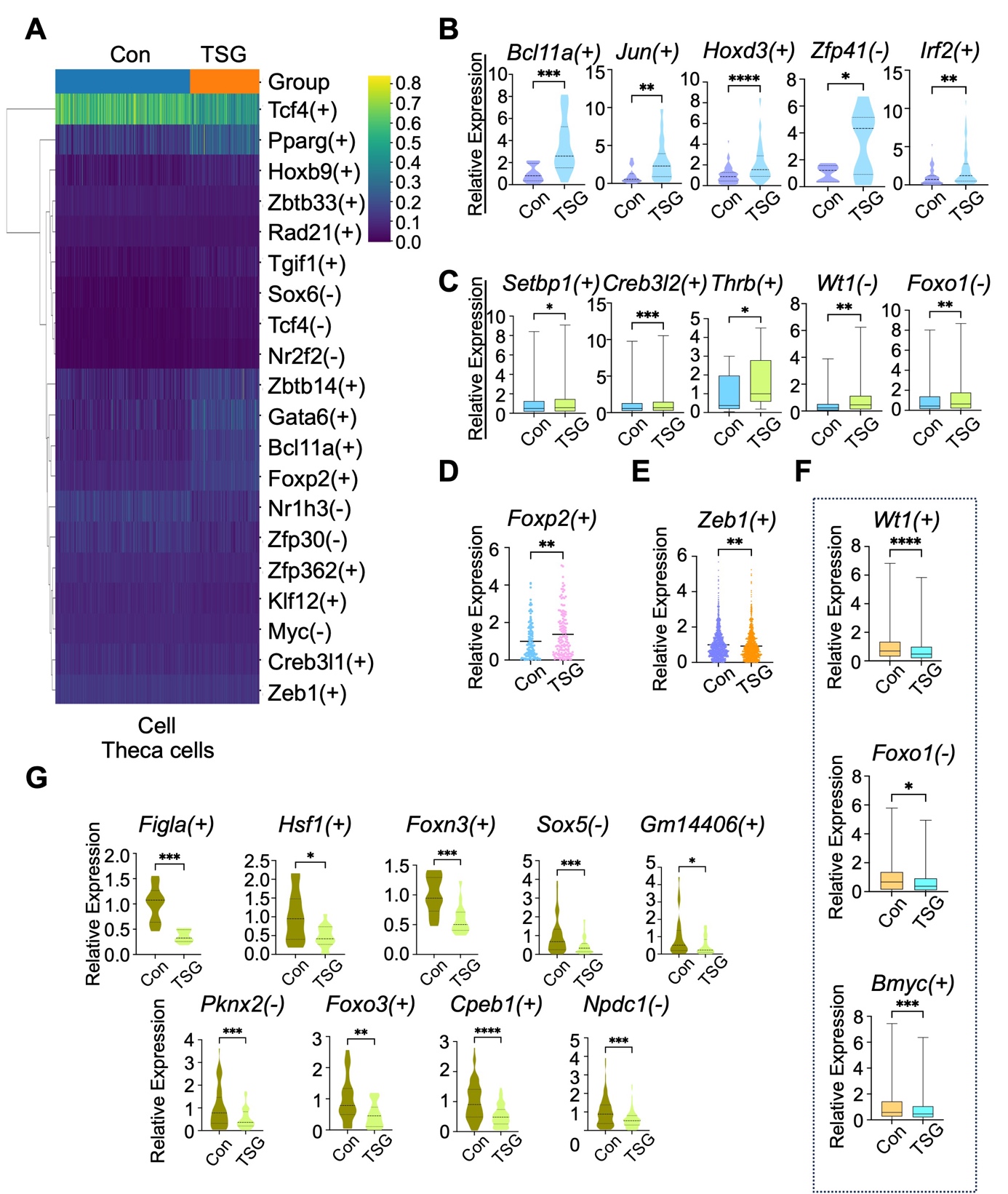


**Figure S6. Transcription factor (TF) regulatory landscape of tetrahydroxy stilbene glucoside (TSG)-treated and control ovarian organoids. A.** Heatmap generated using pySCENIC illustrating TFs activity in theca cells from TSG-treated and control ovarian organoids. **B-D**. Violin plots/panel diagram/scatter plot showing the relative expression of TFs with increased activity in oocytes (B), granulosa cells (GCs) (C), and theca cells (D) from TSG-treated ovarian organoids. **E**-**G**. Scatter plot/ panel diagram/violin plots showing the relative expression of TFs with decreased activity in theca cells (E), GCs (F), and oocytes (G) from TSG-treated ovarian organoids.


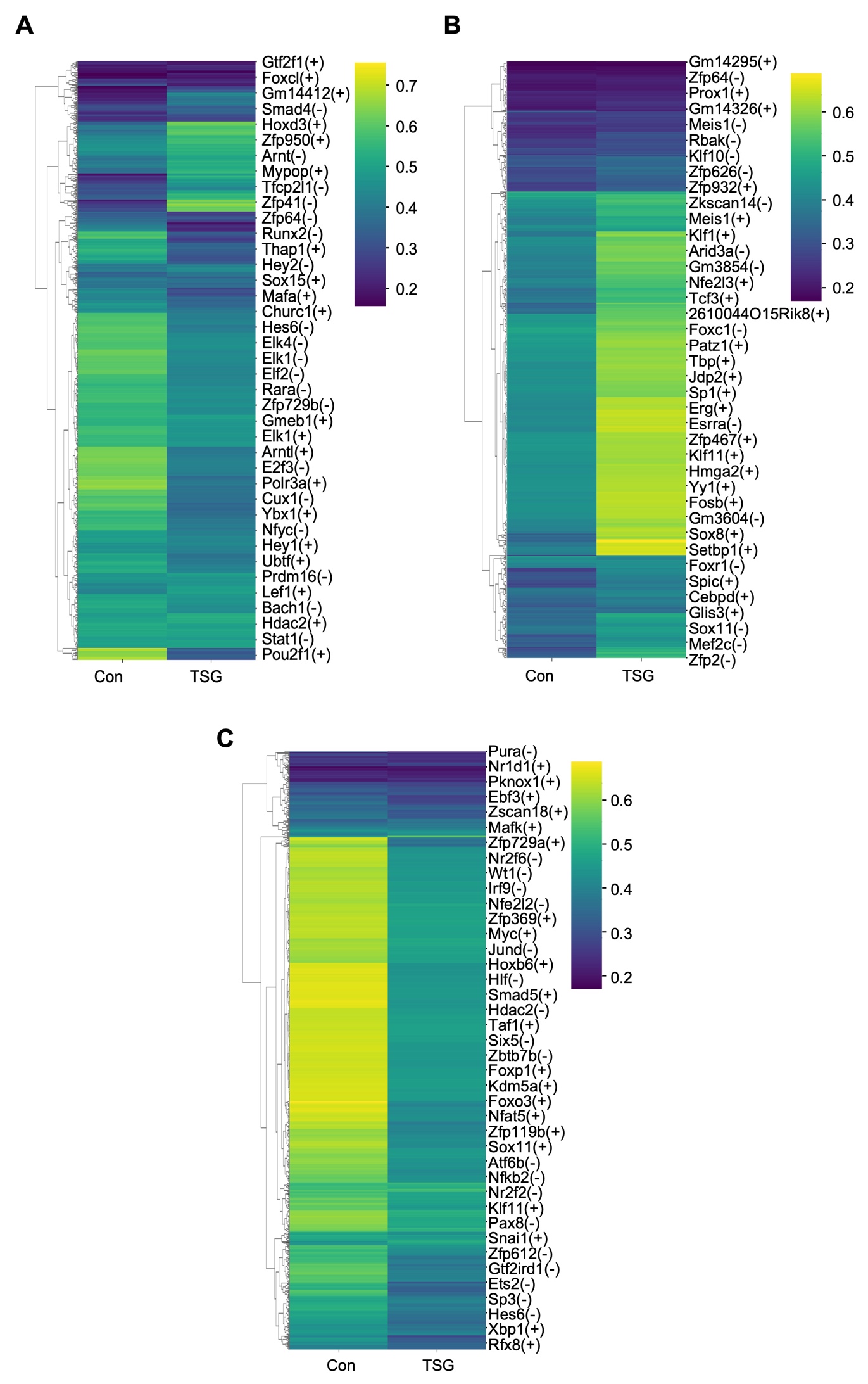


**Figure S7. Transcription factor (TFs) regulatory landscape of tetrahydroxy stilbene glucoside (TSG)-treated and control ovarian organoids. A-C.** Heatmap visualization of upregulated and downregulated specific regulons in oocytes (A), granulosa cells (GCs) (B), and theca cells (C) between TSG-treated and control ovarian organoids.


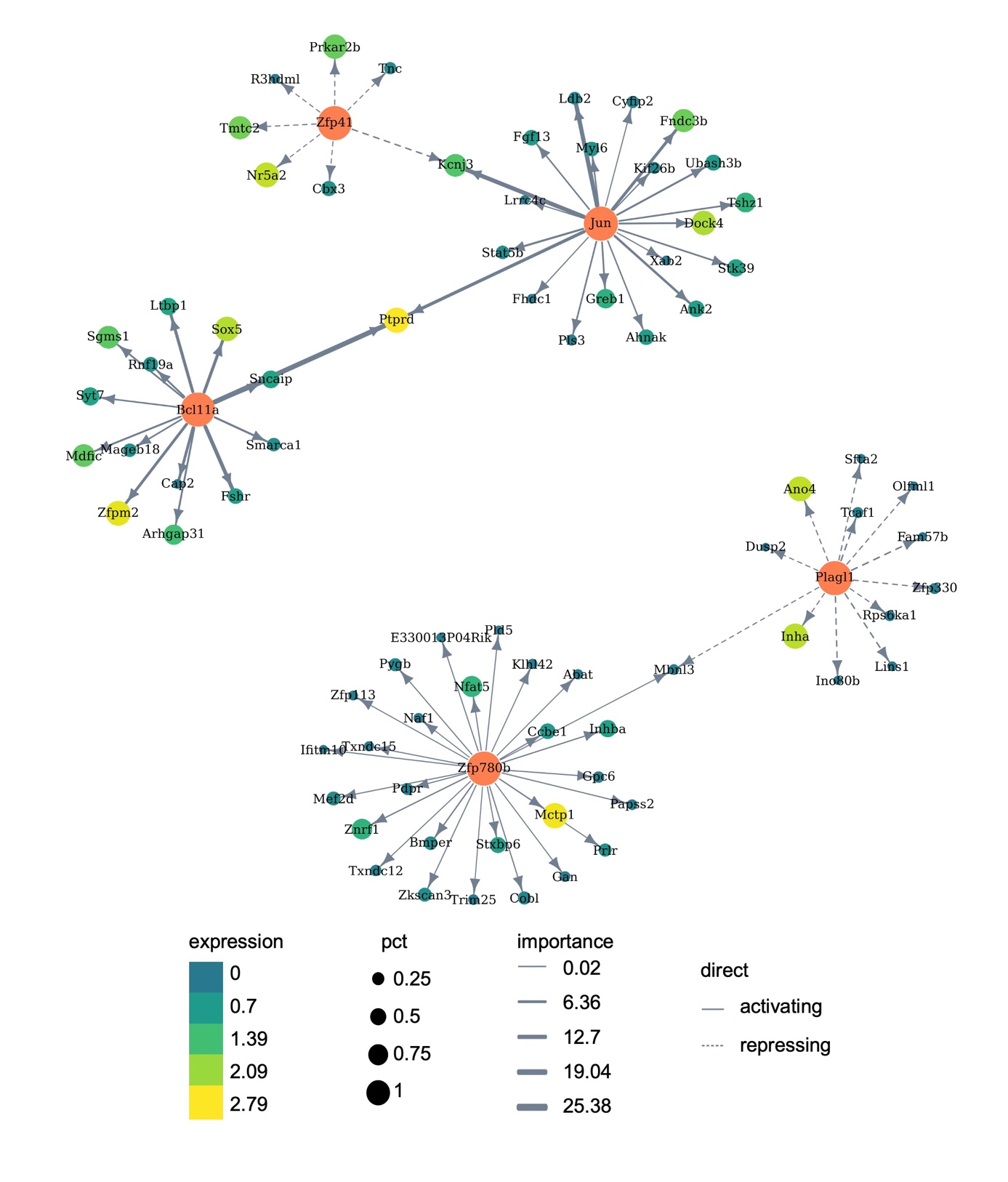


**Figure S8. Gene regulatory network inferred from transcription factors (TFs) dynamically expressed in oocytes in tetrahydroxy stilbene glucoside (TSG)-treated and control ovarian organoids.** The larger central circle represented the TFs, and connected circles denoted target genes. Colors indicate the average expression of target genes. The circle size reflected the percentage of cells expressing the target gene in each cell type. Line thickness represented the influence of TFs on target gene expression, with solid and dashed lines indicating activating and inhibition, respectively.


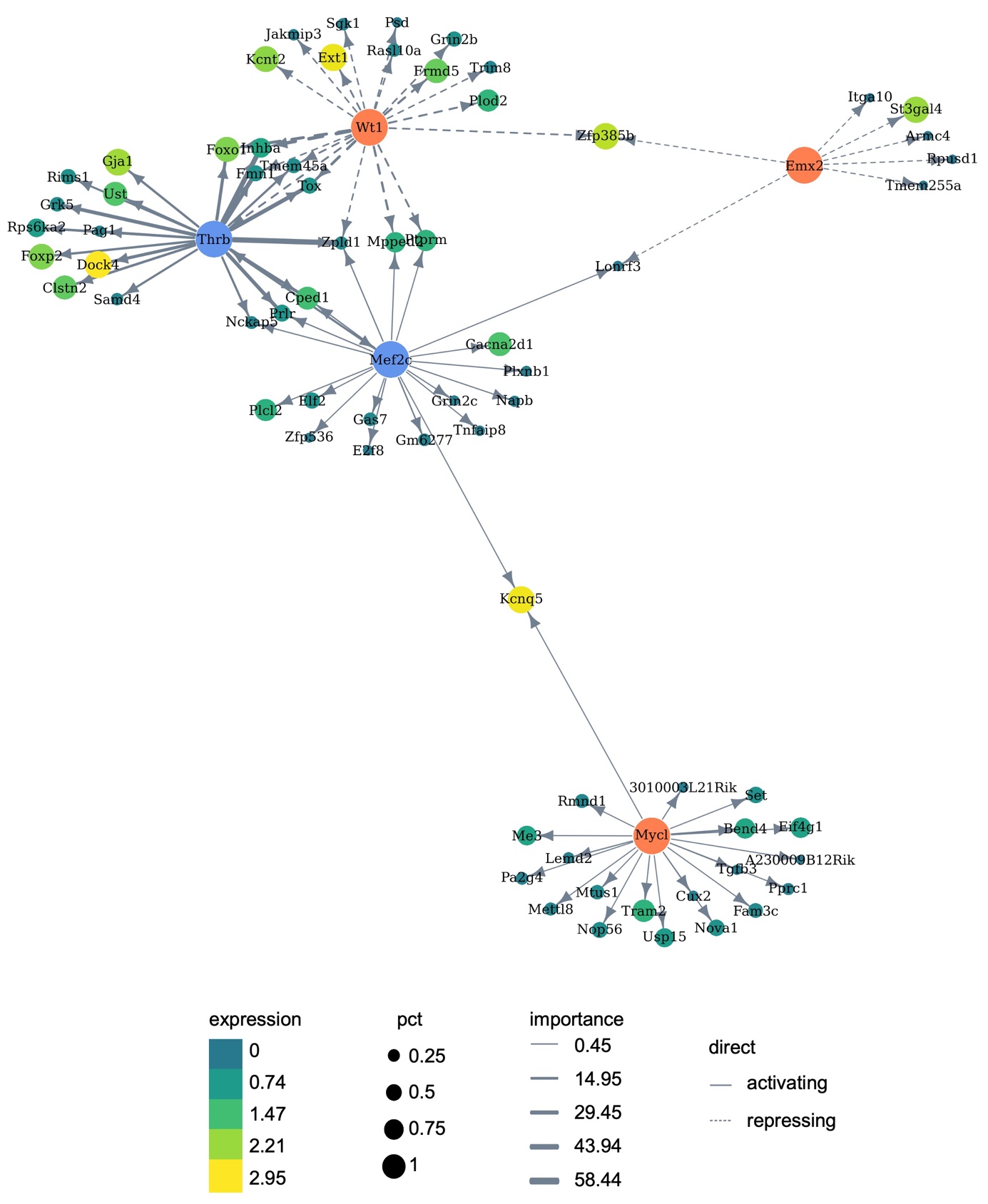


**Figure S9. Gene regulatory network inferred from transcription factors (TFs) dynamically expressed in granulosa cells (GCs) in tetrahydroxy stilbene glucoside (TSG)-treated and control ovarian organoids.** The larger central circle represents the TFs, and connected circles denoted target genes. Colors indicate the average expressions of target genes. The circle size reflected the percentage of cells expressing the target gene in each cell type. Line thickness represented the influence of TFs on target gene expression, with solid and dashed lines indicating activating and inhibition, respectively.


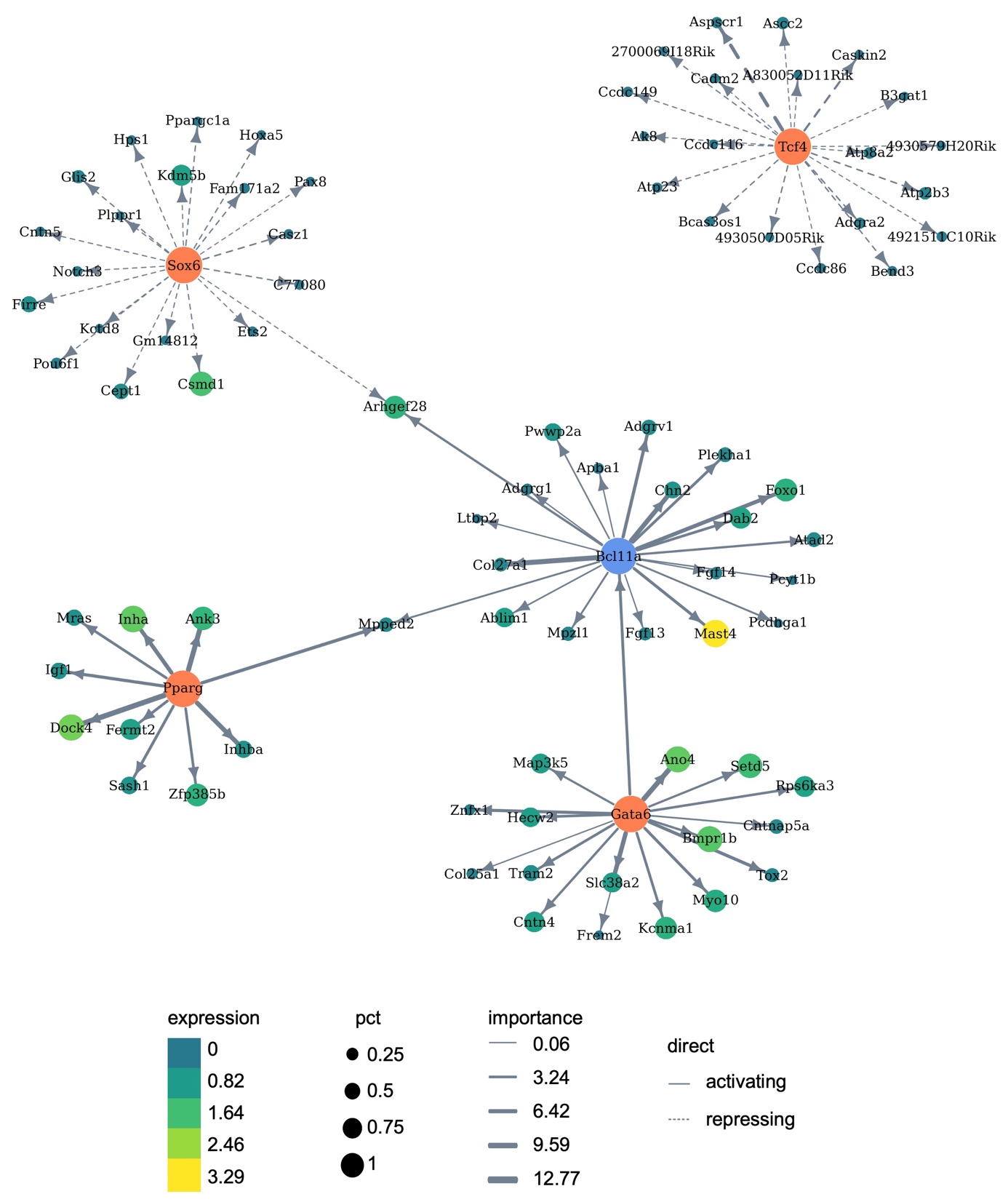


**Figure S10. Gene regulatory network inferred from transcription factors (TFs) dynamically expressed in theca cells in tetrahydroxy stilbene glucoside (TSG)-treated and control ovarian organoids.** The larger central circle represented the TFs, and connected circles denoted target genes. Colors indicated the average expressions level of target genes. The circle size reflects the percentage of cells expressing the target gene in each cell type. Line thickness represents the influence of TFs on target gene expression, with solid and dashed lines indicating activating and inhibition, respectively.


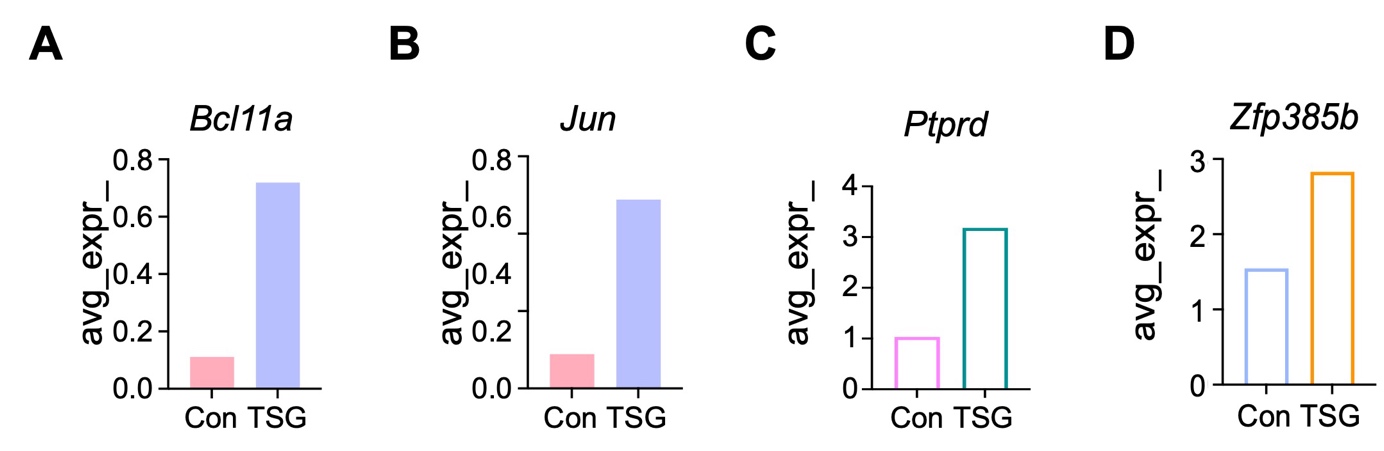


**Figure S11. The expression of selected transcription factors (TFs) and target genes in tetrahydroxy stilbene glucoside (TSG)-treated and control ovarian organoids. A-B.** Bar plots showing the increased expression of TFs in oocytes from TSG-treated ovarian organoids. **C**. Bar plot showing the increased expression of target gene of TFs (*Bcl11a* and *Jun*) in oocytes. **D**. Bar plot showing the increased expression of the target gene of TFs (*Wt1* and *Emx2*) in granulosa cells (GCs).


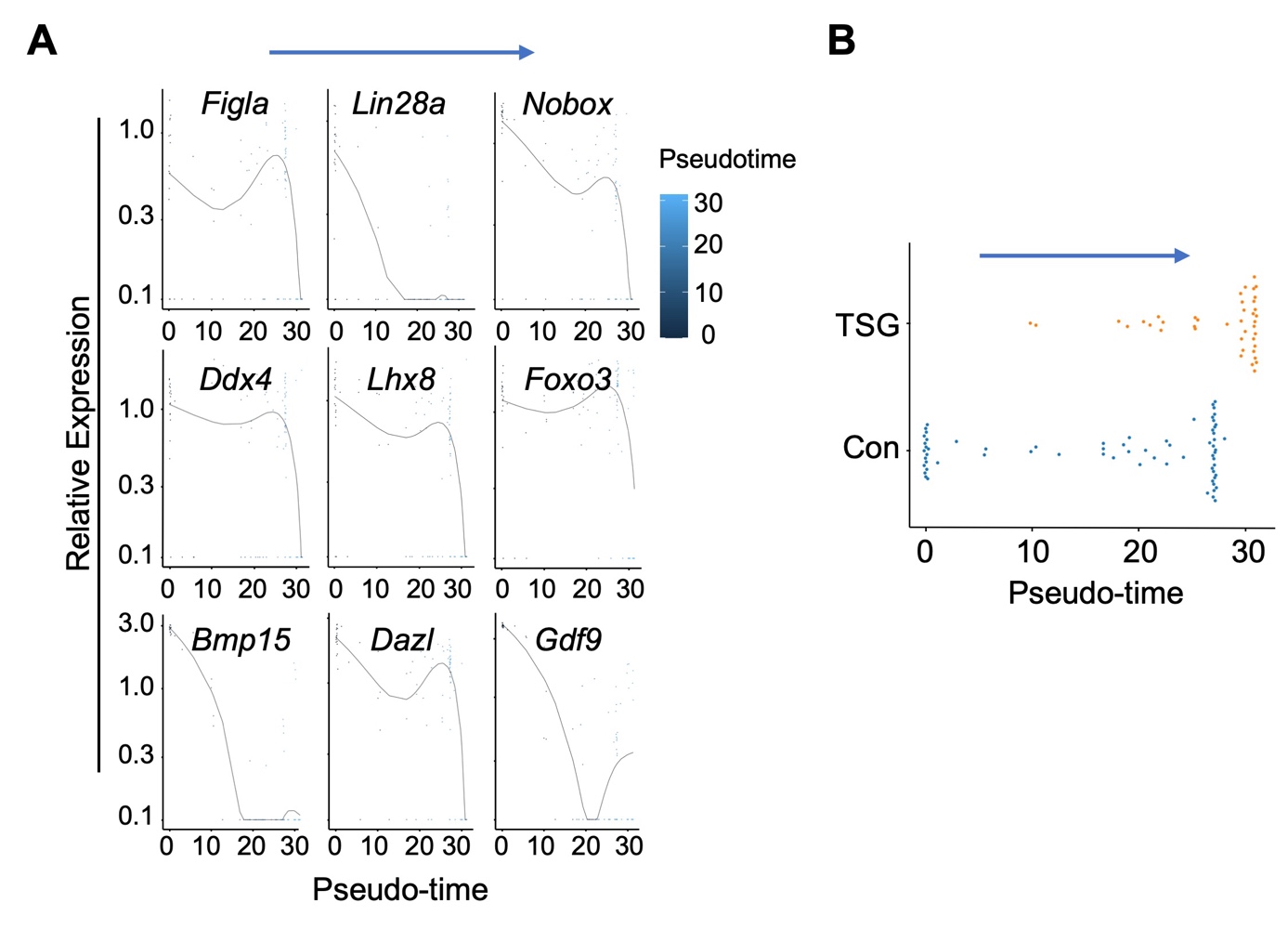


**Figure S12. The cellular transcriptional trajectory during oocyte differentiation in tetrahydroxy stilbene glucoside (TSG)-treated ovarian organoids. A.** Expression of marker genes in oocytes along pseudo-timeline. The black line indicates the expression tendency. **B**. Scatter plot depicting the distribution of oocytes along pseudo-time in TSG-treated and control ovarian organoids.


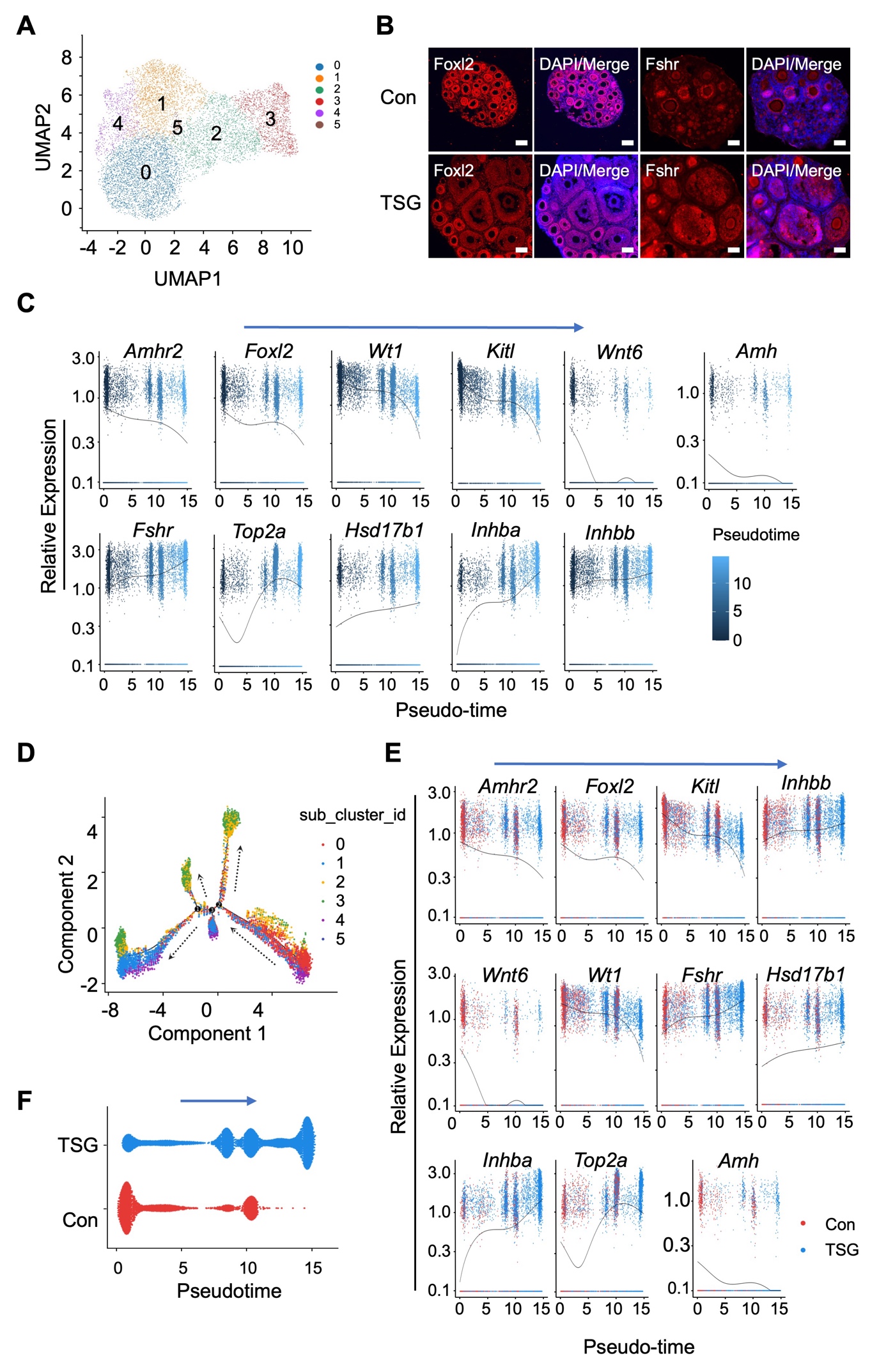


**Figure S13. The cellular transcriptional trajectory during granulosa cell (GC) differentiation in tetrahydroxy stilbene glucoside (TSG)-treated ovarian organoids. A.** The Uniform manifold approximation and projection (UMAP) visualization identified GCs subcluster in ovarian organoids. **B**. Immunofluorescence analysis for GCs markers (Foxl2 and Fshr) in control and TSG-treated ovarian organoids. Nuclei were counterstained with DAPI. Scal bars: 100 μm. **C.** Expression of marker genes in GCs along the pseudo-timeline. The black line indicates the expression tendency. **D**. Pseudo-time trajectory of GCs subclusters along pseudo-time trajectory. **E**. Expression trends of genes in GCs involved in control and TSG-treated ovarian organoids along pseudo-time. **F**. Scatter plot depicting the distribution of GCs along pseudo-time in control and TSG-treated ovarian organoids.


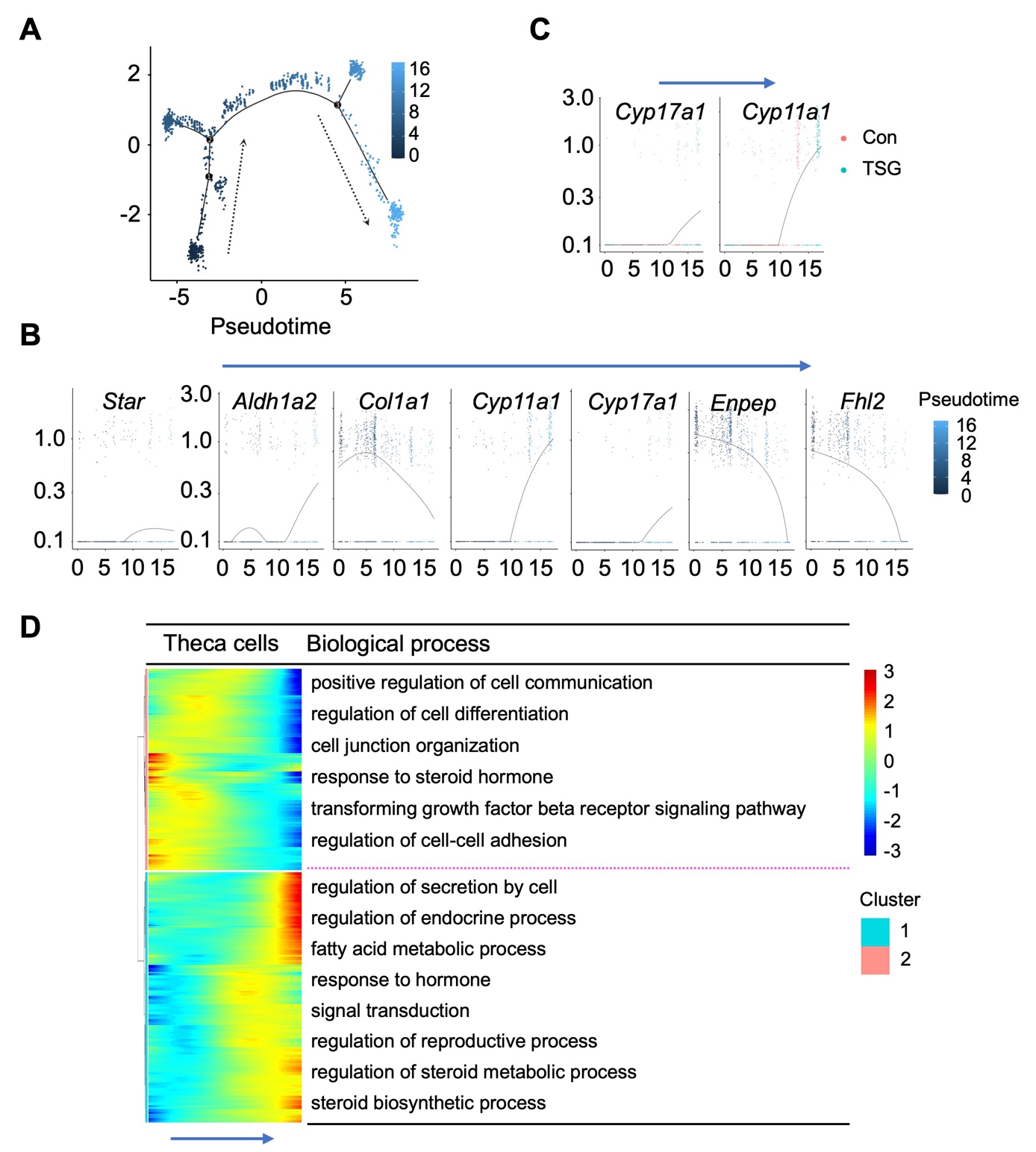


**Figure S14. The cellular transcriptional trajectory during theca cells differentiation in tetrahydroxy stilbene glucoside (TSG)-treated ovarian organoids. A.** Heatmap illustrating two gene modules of significant differentially expressed genes (DEGs) and enriched Gene Ontogeny (GO) biological process in theca cells along the pseudo-time. **B**. Expression of marker genes in theca cells along the pseudo-time. The black line indicates the expression tendency. **C**. Expression trends of genes in theca cells involved in control and TSG-treated ovarian organoids along the pseudo-time. **D**. Heatmap illustrating two gene modules of significant DEGs and enriched GO biological process in theca cells along pseudo-timeline.


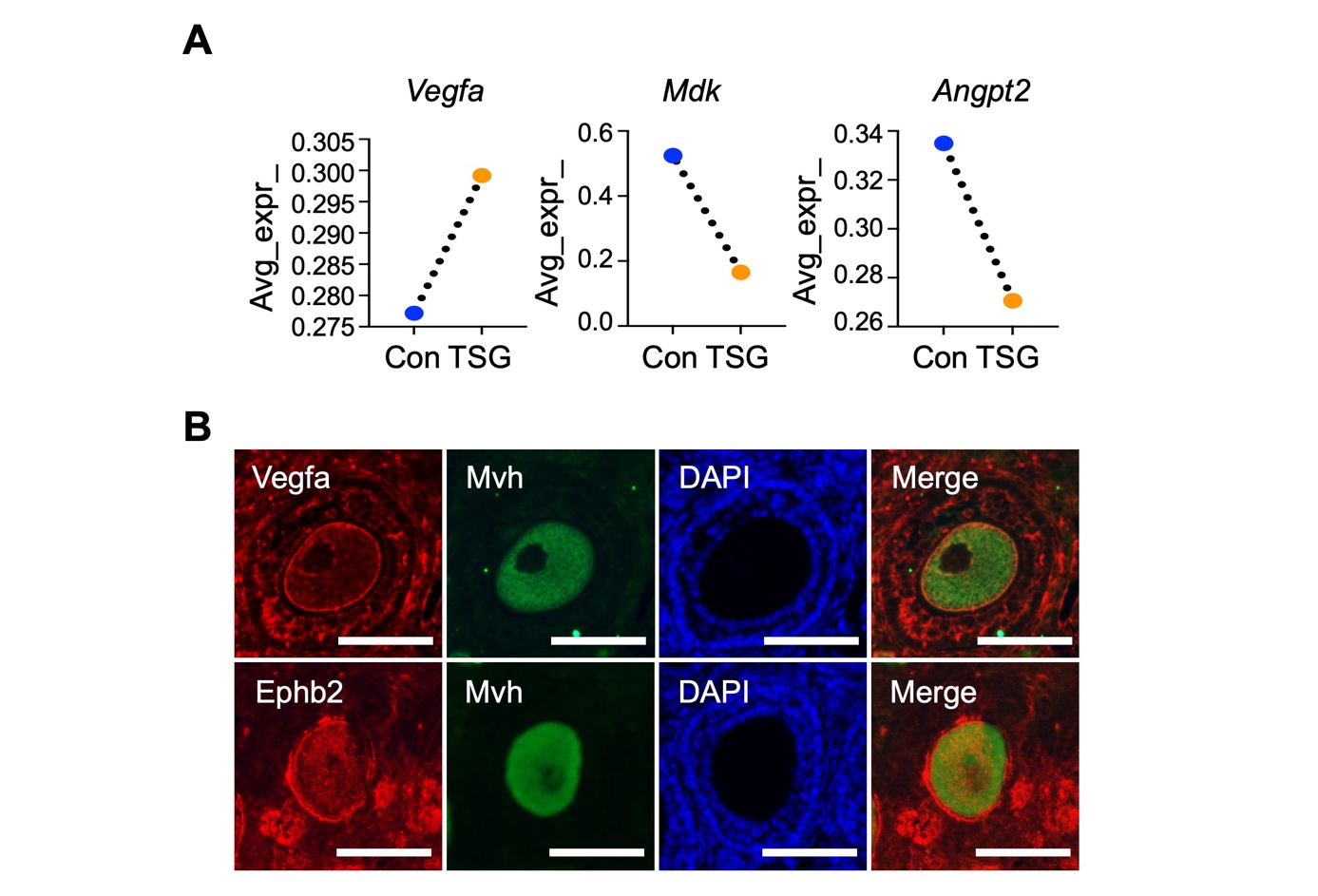


**Figure S15. Tetrahydroxy stilbene glucoside (TSG) promotes ovarian organoid development by mediating the interaction of the Vegfa-Ephb2 pair between granulosa cells (GCs) and oocytes.**

**A.** Single nucleus transcriptome sequencing (snRNA-seq) revealing the gene expression of selected ligands in control and TSG-treated ovarian organoids. **B**. Immunofluorescence analysis of ligand (Vegfa) and receptor (Ephb2) expression in ovarian organoids. The upper images displayed the expression of Vegfa (red) co-stained with the germ cell marker Mvh (green). The bottom images displayed the expression of Ephb2 (red) co-stained with the germ cell marker Mvh (green). Nuclei were stained with DAPI. Scale bars: 100 μm.


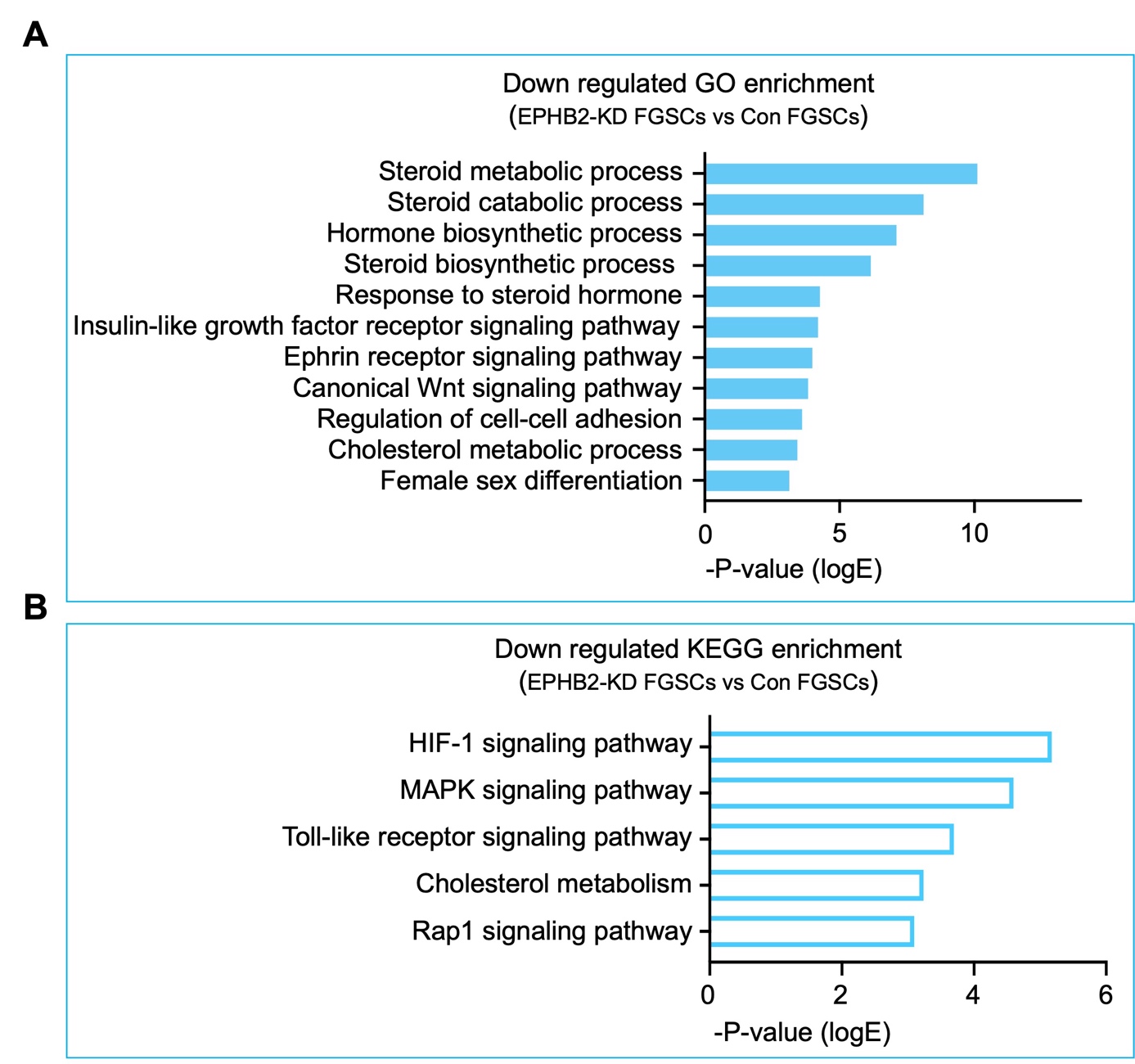


**Figure S16.** **Gene Ontogeny (GO) and Kyoto Encyclopedia of Genes and Genomes (KEGG) enrichment analysis of Ephb2-knock down (KD) female germline stem cells (FGSCs) compared with control.** **A**. Significantly downregulated GO terms (biological processes) identified in differentially expressed genes (DEGs) in Ephb2-KD FGSCs. **B.** Significantly downregulated signaling pathways identified in DEGs in Ephb2-KD FGSCs.

**Table S1 Primers for RT-PCR and RT-qPCR**

| **Primer name** | **Primer sequence (5'-3')** |
| --- | --- |
| *Mvh* F | GCCAGAGGGCTTGATATTGA |
| *Mvh* R | CAACTGGATTGGGAGCTTGT |
| *Oct* F | GTTCAGCCAGACCACCATCT |
| *Oct* R | TGGGAAAGGTGTCCCTGTAG |
| *Dazl* F | AATGACGTGGATGTGCAGAAG |
| *Dazl* R | ACAGTTGTATAAGCCTGGTAGTT |
| *Fragilis* F | AGCCTATGCCTACTCCGTGA |
| *Fragilis* R | GGGTGAAGCACTTCAGGACC |
| *Stella* F | ACCATCAGAGAAAGTCGACCC |
| *Stella* R | AATGGCTCACTGTCCCGTTC |
| *Sycp3* F | ATGATGGAAACTCAGCAGCAAGAGA |
| *Sycp3* R | TTGACACAATCGTGGAGAGAACAAC |
| *Gdf9* F | TCTTAGTAGCCTTAGCTCTCAGG |
| *Gdf9* R | TGTCAGTCCCATCTACAGGCA |
| *Zp3* F | ATGGCGTCAAGCTATTTCCTC |
| *Zp3* R | CGTGCCAAAAAGGTCTCTACT |
| *Ephb2* F | GCGGCTACGACGAGAACAT |
| *Ephb2* R | GCGGCTACGACGAGAACAT |
| *Vegfa* F | CTGCCGTCCGATTGAGACC |
| *Vegfa* R | CCCCTCCTTGTACCACTGTC |
| *Gapdh* F | AGGTCGGTGTGAACGGATTTG |
| *Gapdh* R | TGTAGACCATGTAGTTGAGGTCA |

**Table S1. Primers for RT-PCR and RT-qPCR.**
